# Interactions of Globular and Ribbon [γ4E]GID with α4β2 Neuronal Nicotinic Acetylcholine Receptor

**DOI:** 10.3390/md19090482

**Published:** 2021-08-26

**Authors:** Xiaosa Wu, David J. Craik, Quentin Kaas

**Affiliations:** 1 Institute for Molecular Bioscience, Australian Research Council Centre of Excellence for Innovations in Peptide and Protein Science, The University of Queensland, Brisbane, QLD 4072, Australia; xiaosa.wu@nih.gov; 2 National Institutes of Health, Building 35A, Room 3D-953B, 35 Convent Drive, Bethesda, MD 20892-3701, USA

**Keywords:** α4β2 nAChR, α-conotoxin, molecular dynamics simulation, FoldX, drug design

## Abstract

The α4β2 nAChR is implicated in a range of diseases and disorders including nicotine addiction, epilepsy and Parkinson’s and Alzheimer’s diseases. Designing α4β2 nAChR selective inhibitors could help define the role of the α4β2 nAChR in such disease states. In this study, we aimed to modify globular and ribbon α-conotoxin GID to selectively target the α4β2 nAChR through competitive inhibition of the α4(+)β2(−) or α4(+)α4(−) interfaces. The binding modes of the globular α-conotoxin [γ4E]GID with rat α3β2, α4β2 and α7 nAChRs were deduced using computational methods and were validated using published experimental data. The binding mode of globular [γ4E]GID at α4β2 nAChR can explain the experimental mutagenesis data, suggesting that it could be used to design GID variants. The predicted mutational energy results showed that globular [γ4E]GID is optimal for binding to α4β2 nAChR and its activity could not likely be further improved through amino-acid substitutions. The binding mode of ribbon GID with the (α4)_3_(β2)_2_ nAChR was deduced using the information from the cryo-electron structure of (α4)_3_(β2)_2_ nAChR and the binding mode of ribbon AuIB. The program FoldX predicted the mutational energies of ribbon [γ4E]GID at the α4(+)α4(−) interface, and several ribbon[γ4E]GID mutants were suggested to have desirable properties to inhibit (α4)_3_(β2)_2_ nAChR.

## 1. Introduction

The α4β2 nAChR is the most abundant and widely distributed neuronal nAChR and is a potential target for a range of neurological conditions and disorders [1], including nicotine addiction [2,3], epilepsy [4], Parkinson’s and Alzheimer’s disease [5]. The heteromeric α4β2 nAChR has two major stoichiometries with distinct functional properties: (α4)_2_(β2)_3_ (a pentamer with two α4 subunits and three β2 subunits) and (α4)_3_(β2)_2_ (a pentamer with three α4 subunits and two β2 subunits) [6,7]. The two subtypes differ in their sensitivity to agonists, antagonists and allosteric modulators. Each subtype also has distinct single-channel conductance [8], mean open lifetime and activation-deactivation kinetics [9]. Both stoichiometries are associated with nicotine addiction and congenital epilepsy [10,11]. Previous studies showed that chronic inactivation of α4β2 nAChR could result in significant impairment of spatial memory in rodents [12,13], indicating that understanding α4β2 nAChR antagonists could assist in designing drugs to treat memory impairment.

**Figure 1:**
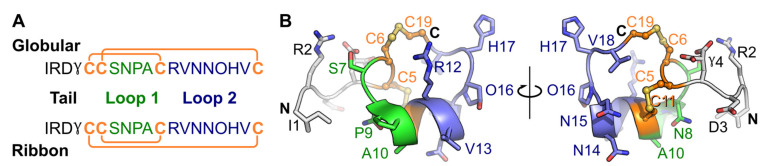
Amino acid sequence of globular and ribbon GID (**A**) and the three-dimensional NMR solution structure of gGID (PDB: 1MTQ) (**B**). γ: γ-carboxyglutamic acid, O: hydroxyproline; the connectivities of disulfide bonds are shown as orange lines and the disulfide bonds are shown as orange sticks in the 3D structure; “C” and “N” stand for the C-terminus and N-terminus, respectively. The two loops, which are defined as inter-Cys segments, are shown in green and blue, respectively, on the amino acid sequence and three-dimensional structure. In panel B, the globular GID three-dimensional structure is shown in two orientations differentiated by a 180° turn along the indicated axis.

To date, no conotoxin that can selectively and potently block the α4β2 subtype has been discovered [14]. α-Conotoxins contain four cysteine residues forming two disulfide bonds, which can generate three isomers [15]: globular (Cys I-Cys III and Cys II-Cys IV), ribbon (Cys I-Cys IV and Cys II-Cys III), or bead (Cys I-Cys II and Cys III-Cys IV). Some globular α-conotoxins exhibit low inhibition of the α4β2 receptor, suggesting that it is possible to target this receptor with a peptide [16,17,18,19]. These conotoxins are nevertheless more active at other nAChR subtypes and need to be optimized to generate selective compounds for the α4β2 nAChR. Unusually amongst conotoxins, α-conotoxin GID (Figure 1), discovered from *Conus geographus*, has a four-residue N-terminal tail and residues with post-translational modifications in positions 4 and 16 [17]. Globular GID (gGID) inhibits the rat α3β2, α4β2 and α7 nAChRs with IC_50_ values of 3.4 nM, 128.6 nM and 5.1 nM, respectively [20]. An Ala scan of non-cysteine residues showed that most gGID Ala mutants had at least a 10-fold decrease in activity at the α4β2 nAChR [20]. Banerjee et al. built a binding mode of gGID with an α4(+)β2(−)interface using the crystal structure of the acetylcholine binding protein (AChBP) in complex with [A10L, D14K]PnIA as a template, which was used to design new mutants to more selectively and potently inhibit α4β2 nAChR. Of 22 mutants that were designed and tested, [V18N]gGID maintained comparable activity at the rat α4β2 nAChR but had no activity at the rat α3β2 nAChR, suggesting that the selectivity against different subtypes of this molecule can be improved using a de novo design [21]. Likewise, Leffler et al. built a molecular model of gGID interacting with the α4(+)β2(−) interface based on the crystal structures of AChBPs in complex with α-conotoxins [A10L, D14K]PnIA, ImI, BuIA and [A10L]TxIA, which was refined using docking algorithm ToxDock based on the Rosetta modeling framework [22]. The refined molecular model was then used to virtually screen some predicted bioactive peptides selected to evaluate their activities against a range of nAChRs. The [V13Y]gGID displayed reduced activity at the human α7 nAChR from 0.1 µM to 4 µM while maintaining the same activity at the α4β2 nAChR (3 µM and increased activity at the α3β2 nAChR from 10 nM to 2 nM [22].

Rational modification of gGID to selectively and potently block α4β2 nAChRs has been attempted but did not succeed, possibly due to imperfect models of the GID/α4β2 complex. Although the AChBP is a structural homologue to nAChRs and is typically considered as a structural surrogate of the nAChR ligand-binding domain, low sequence identity (<30%) prevents the construction of a reliable α4β2 nAChR molecular model using homology modeling only. Recently, the high-resolution 3D structures of (α4)_2_(β2)_3_ and (α4)_3_(β2)_2_ nAChRs have been reported [23,24], providing an opportunity to build a more accurate molecular model of the complex between GID and α4β2 nAChR. The globular [γ4E]GID ([γ4E]gGID) mutant was reported to have comparable activity at rat α3β2, α4β2 and α7 nAChRs to gGID [20], and this variant was used to build the molecular models described below. [γ4E]gGID is referred to as “gGID*” hereafter and likewise, ribbon [γ4E]GID is referred to as “rGID*”.

Here, we report on molecular modeling and energy calculations for complexes between gGID*/rGID* and several nAChR subtypes, which could be used to develop selective and potent α4β2 nAChR inhibitors. The binding modes of gGID* at α3β2, α4β2 and α7 nAChRs were modeled at five interfaces: α3(+)β2(−), α3(+)α3(−), α4(+)β2(−), α4(+)α4(−) and α7(+)α7(−) interfaces. As noted in our previous study [25], ribbon α-conotoxins can bind at the α(+)α(−) interface, suggesting that rGID could bind the (α4)_3_(β2)_2_ nAChR at the α4(+)α4(−) interface. We derived the binding mode of ribbon α-conotoxin rAuIB at the α3(+)α3(−) binding site using molecular dynamics simulations oriented by experimental activity changes for Ala substitution in rAuIB [25]. Our investigations suggested that ribbon α-conotoxins adopt a shape that is more compatible with an interaction at the α/α interface rather than at the α/β interface. We therefore built molecular models of the interaction between rGID* and α3(+)α3(−), α4(+)α4(−) or α7(+)α7(−) binding sites using the rAuIB/α3(+)α3(−) model as a template, with the aim of modifying the peptide to selectively and potently inhibit (α4)_3_(β2)_2_ nAChR. The models were then used to calculate mutational energies (ΔΔG) of GID* mutants and to suggest potential α4β2 nAChR inhibitors.

## 2. Results

### 2.1. Binding Modes of GID*

We developed molecular models to evaluate the possibility of developing a selective inhibitor of α4β2 nAChR. Molecular models of the complexes between gGID* and three nAChR subtypes from rat were first built and validated by analyzing available structure-activity-relationships data. We also built alternative models of interactions between rGID* and human nAChR subtypes. There is no mutational data available at the human subtypes for neither gGID* or rGID*, but developing inhibitors of human α4β2 nAChR is the overarching goal.

We built molecular models of the interaction between gGID* and the α4(+)β2(−), α4(+)α4(−), α3(+)β2(−), α3(+)α3(−) or α7(+)α7(−) binding sites as well as between rGID* and the α4(+)α4(−), α3(+)α3(−) and α7(+)α7(−) binding sites. These models were refined using molecular dynamics simulations and used to propose explanations for the SAR data reported by Millard et al. [20]. The backbone root-mean-square deviation from the starting conformation was stable over the last 20 ns of the simulations, and this period was referred to as ‘simulation time’ and was used for analysis. The binding modes of gGID* and rGID* were similar in all modeled binding sites (Figure S1). The N-terminal tail of gGID*, corresponding to the four residues at the N-terminus of gGID*, was found to be the most flexible, but its conformational space is sterically constrained by the C- and F-loops (illustrated in the α4(+)β2(−) binding site in Figure S2).

#### 2.1.1. Binding Modes of gGID* at the Rat α4(+)β2(−) and α4(+)α4(−) Interfaces

**Figure 2:**
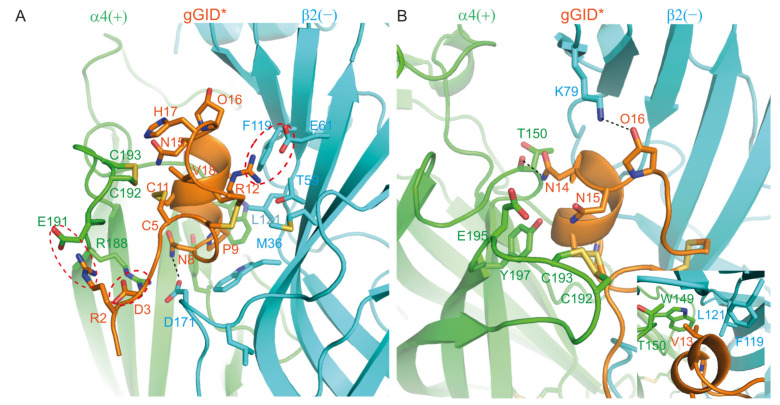
Model of the complex between gGID* and the α4(+)β2(−) interface. (**A**) Interactions between the receptor and Arg-2 (R2), Asp-3 (D3), Asn-8 (N8), Pro-9 (P9), Arg-12 (R12), His-17 (H17) and Val-18 (V18) of gGID*; (**B**) Interactions between the receptor and Val-13 (V13) (insert panel), Asn-14 (N14), Asn-15 (N15) and Hyp-16 (O16) of the gGID*. Hydrogen bonds are displayed as dotted black lines. Interactions between charged side chains are circled with a dotted red line.

The SAR experimental data for gGID* in a previous study showed that R2A, D3A, N8A, P9A, R12A, N14A, N15A, O16A, H17A and V18A mutants lost activity at α4β2 nAChR by more than 8-fold [20]. The model of the complex between gGID* and the α4(+)β2(−) binding site can be used to provide rational explanations for this data (Figure 2).

In the proposed model, Arg-2 and Asp-3 form salt bridges with α4 (+)-E191 and α4 (+)-R188, respectively. Therefore, substitution of these two residues by Ala could prevent the establishment of the salt bridges, potentially resulting in a change of conformation of the tail, which could explain why the substitution of these two residues by Ala caused a decrease in activity >18-fold [20]. Pro-9 (corresponding to Pro-6 in most other α-conotoxins) is highly conserved in all α-conotoxins and is crucial for stabilizing the structure of globular α-conotoxins; substitution of Pro by an Ala typically results in a dramatic decrease in activity [26,27,28,29,30,31,32,33,34]. In our model, Arg-12 is buried at the interface with the receptor and forms a salt bridge with β2 (−)-E61. Its side-chain atoms are located within 5 Å of the M36, T59, E61, F119 and L121 of the β2 (−) subunit (a distance <5 Å is considered to be a contact in this study). It is therefore not surprising that substitution of Arg by Ala caused a decrease in activity of >18-fold [20]. Residues Asn-8, Asn-14 and Hyp-16 established hydrogen bonds with β2 (−)-D171, α4 (+)-T150 and β2 (−)-K79, respectively. These interactions were present in 85%, 94% and 71% of the molecular dynamics simulation time, respectively. These hydrogen bonds seem to be crucial for the activity of the peptide as experimental data showed that the loss of any of these three hydrogen bonds caused a significant drop in activity [20]. In our model, Asn-15 interacts with the C-loop disulfide bond (formed by α4 (+)-C192 and α4 (+)-C193), E195 and Y197. His7 contacts the C-loop disulfide bond. The sidechain of Val-18 is embedded into a pocket formed by Cys-5, Cys-6, Arg-12, Asn-15, Hyp-16 and His-17 of gGID* as well as the C-loop disulfide bond. Substitutions of Asn-15, His-17 and Val-18 by Ala could reduce the affinity of the peptide for nAChRs, resulting in a decrease in activity. In agreement with the experimental data, the N15A, H17A and V18A mutants demonstrate reduced activity compared to the parent peptide [20]. In the molecular model, the side chain of Val-13 contacts α4 (+)-W149, α3 (+)-T150, β2 (−)-F119 and β2 (−)-L121, and substitution of Val-13 by Ala should result in less contact between the peptide and the α4β2 nAChR, negatively impacting the activity. By contrast, the experimental data show that the V13A mutant has comparable activity to the gGID*, suggesting that interpreting the mutational data by side-chain interactions is not sufficient, as reorientation can occur in the binding pocket of the conotoxin after a side chain replacement.

We also modeled the interactions in the α4(+)α4(−) binding site, which are illustrated in Figure S3. The binding mode was very similar to that in the α4(+)β2(−) binding site but a few interactions differ even with the shared α4(+) subunit. For example, the salt bridge between Asp-3 and R188 was less frequently observed. Most of the differences occur at the interface with the complementary subunit; the side chain of His-17 has a charge-charge interaction with that of E59, and Arg-12 forms a buried salt-bridge with D122. There is a “void” volume at the interface with the complementary subunit, which does not occur in the α4(+)β2(−) binding site, suggesting that the shape complementarity is not as optimal in the α4(+)α4(−) binding site compared to the α4(+)β2(−) binding site.

#### 2.1.2. Binding Modes of gGID* at the Rat α3(+)β2(−) and α3(+)α3(−) Interfaces

**Figure 3:**
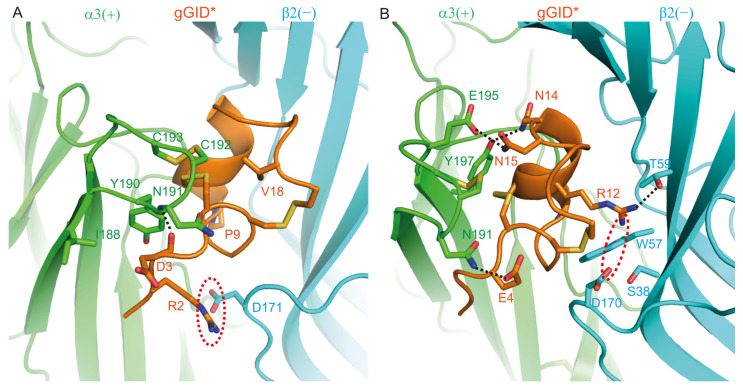
Model of the complex between gGID* and the α3(+)β2(−) interface. (**A**) Interactions between the receptor and Arg-2 (R2), Asp-3 (D3), Pro-9 (P9) and Val-18 (V18) of gGID*; (**B**) Interactions between the receptor and Glu-4 (E4), Arg-12 (R12), Asn-14 (N14) and Asn-15 (N15) of the gGID*. Hydrogen bonds are displayed as dotted black lines. Interactions between charged side chains are circled with a dotted red line.

The R2A, D3A, P9A and V18A mutants display decreased (>8-fold) potency at the rat α3β2 nAChR compared to gGID* [20]. Our model (Figure 3A) suggests that a salt bridge is formed between the Arg-2 and D171 of the β2(−) subunit. The oxygen of Asp3 forms a stable hydrogen bond (99% of the simulation time) with the nitrogen of the α3(+)-N191 as well as consistent contact with the I188, Y190 and N191 of the α3(+) subunit. The side chain of Val18 interacts with the disulfide bond of the C-loop.

In the reported SAR study, the E4A, R12A, N14A and N15A mutants had no change in activity compared to gGID* [20]. However, our model (Figure 3B) shows that Glu-4 establishes a hydrogen bond with α3(+)-N191, which is present during 98% of the simulation time. The Arg-12 side chain forms an unstable hydrogen bond with β2(−)-T59 for 41% of the simulation time and has a charge interaction with β2(−)-D170. It also contacts S38, W57 and T59 of the β2-(−) subunit. Asn-14 forms hydrogen bonds with α3 (+)-Y197 (94% of the simulation time) and Asn-15 forms a hydrogen bond with α3(+)-E195 (99% of the simulation time).

The interactions of gGID* in the α3(+)α3(−) binding site are illustrated in Figure S3. In that model, Arg-12 establishes a salt-bridge interaction with E34 from the complementary subunit but is also proximal to the positively charged K57, which is buried at the interface and forms a hydrogen bond with the backbone carbonyl of Arg-12. The interface with the complementary subunit is well-packed, suggesting a good shape complementarity. His-17 seemed to establish an attractive charge interaction with E115. The side chain of Asn-14 established a network of hydrogen bonds with the side chains of R79 and Y197.

#### 2.1.3. Binding Modes of gGID* at the Rat α7(+)α7(−) Interface

**Figure 4:**
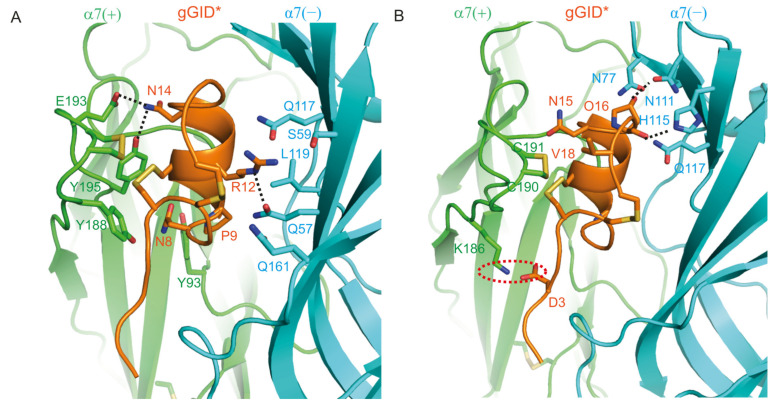
Molecular model of the complex between gGID* and the α7(+)α7(−) interface. (**A**) Interactions between the receptor and Asn-8 (N8), Pro-9 (P9), Arg-12 (R12) and Asn-14 (N14) of gGID*; (**B**) Interactions between the receptor and Asp-3 (D3), Asn-15 (N15), Hyp-16 (O16) and Val-18 (V18) of gGID*. Hydrogen bonds are displayed as dotted black lines. Interactions between charged side chains are circled with a dotted red line.

Positions 8, 9, 12 and 14 of gGID* are the main determinants of the interaction with the rat α7 nAChR, according to experimental data [20]. In Figure 4A, the model shows that Asn-8 constantly contacts α7 (+)-Y93, α7 (+)-Y188 and α7 (+)-Y195. A hydrogen bond is established between Arg-12 and α7 (−)-Q57 for 79% of the simulation time, and the side chain of Arg-12 interacts with Q57, S59, Q117, L119 and Q161 of the α7 (−) subunit. Asn-14 forms two stable hydrogen bonds with α7 (+)-E193 (85% of the simulation time) and α7 (+)-Y195 (91% of the simulation time).

Apart from the 8, 9, 12 and 14 positions, the experimental substitution of other positions with Ala had only a minor impact on the inhibition of rat α7 nAChR by gGID*. Nevertheless, the model shows that positions 3, 15, 16 and 18 have substantial interactions with the receptor (Figure 4B). Specifically, Asp3 forms a salt bridge with α7 (+)-K186. The Hyp16 forms hydrogen bonds with α7 (−)-N111 and α7 (−)-H115 for 66% of the simulation time as well as contacting α7 (−)-N77 and α7 (−)-Q117. Asn15 and Val18 make contact with the C-loop disulfide bond. As mentioned previously, interpreting side-chain mutations using only the wild-type model is limited. The wild-type model does not compensate for energy loss by conformation change or change of binding mode, which can occur in conotoxins mutated during an alanine scan.

#### 2.1.4. Binding Modes of rGID* at the Human α3(+)α3(−), α4(+)α4(−) and α7(+)α7(−) Interfaces

**Figure 5:**
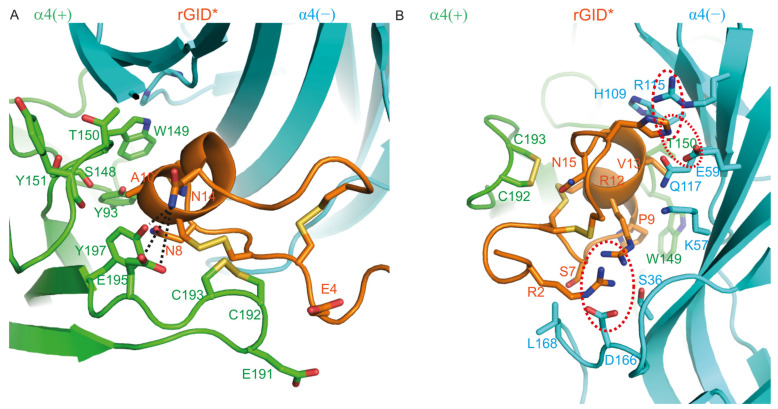
Molecular model of the complex between rGID* and the α4(+)α4(−) interface. (**A**) Interactions between the receptor and Glu-4 (E4), Asn-8 (N8), Ala-10 (A10) and Asn-14 (N14) of rGID*; (**B**) Interactions between the receptor and Arg-2 (R2), Ser-7 (S7), pro-9 (P9), Val-13 (V13), Asn-15 (N15) and His-17 (H17) of rGID*. Hydrogen bonds are displayed as dotted black lines on the structure. Interactions between charged side chains are circled with a dotted red line.

We here propose a model of the interaction of rGID* with the α4(+)α4(−) binding site to inform the design of variants that could bind at this interface. According to this model (Figure 5A,B), the side chain of Arg-2 forms a salt bridge with α4 (−)-D166. However, Arg-2 can also have an electrostatic repulsion with Arg-12 of rGID*. Overall, Arg-2 could thus be used to modulate the activity of rGID* at the α4(+)α4(−) binding site. In loop 1, Glu-4 interacts with the C-loop disulfide bond as well as α4 (+)-E191. Ser-7 and Asn-8 contact the α4 (−)-L168 and α4 (+)-Y93, respectively. As mentioned previously, Pro-9 is an important compact point for globular α-conotoxins as it binds in a conserved aromatic pocket of the nAChR, which is also the binding site of Ach. Ala-10 makes contact with Y93, S148, W149, T150, Y151 and Y197 of the α4 (+) subunit. In loop 2 of rGID*, the sidechain of Arg-12 establishes attractive charge interactions with α4 (−)-D166 and contacts α4 (−)-S36 and K57, but also creates an electrostatic repulsion with Arg-2. The sidechain of Val-13 contacts α4 (+)-Y149, α4 (+)-T150, α4 (−)-H109, α4 (−)-Q117, α4 (−)-W118 and α4 (−)-T119. Asn-14 forms a transient hydrogen bond with α4 (+)-E195 (42% of the simulation time) and a stable hydrogen bond with α4 (+)-Y197 (73% of the simulation time). Asn-15 sits in a pocket formed by three disulfide bonds (disulfide bond of C-loop and two disulfide bonds of rGID*), which seems to stabilize the conformation of the second loop of rGID* at the binding site. If His-17 is positively charged, then it could display a complementary charge to α4 (−)-Glu-59, but in that case would create a charge repulsion with the side chain of α4 (−)-R115, which is mainly solvated and is distant from the binding site in our models.

The interactions of rGID* with α3(+)α3(−) or α7(+)α7(−) interfaces are illustrated in Figure S3. These models display a lower number of hydrogen bonds at the interface compared to those involving gGID*, suggesting that the interface could be improved through mutations. More details will be provided in the next section on mutational energy predictions and amino acid scan of gGID* and rGID*.

### 2.2. ΔΔGs Prediction

#### 2.2.1. Full Sequence Amino Acid Scanning of gGID* at Rat α3β2, α4β2 and α7 nAChR

**Figure 6:**
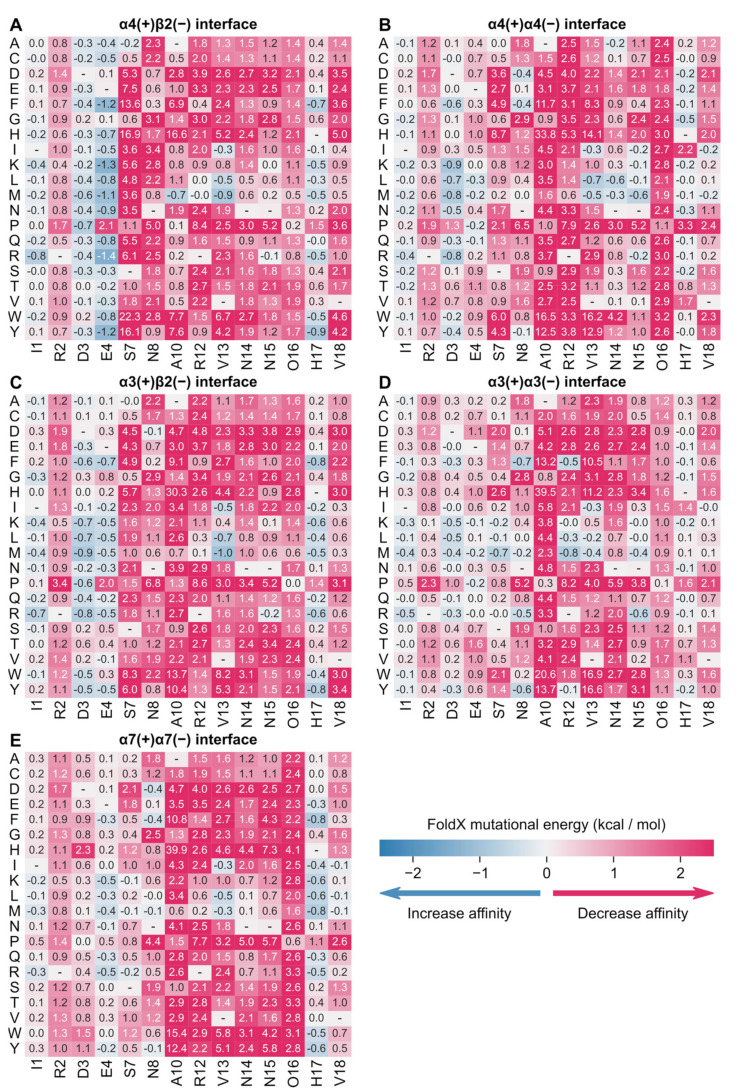
Mutational energies of gGID* predicted by FoldX in the context of five binding sites: (**A**) α4(+)β2(−), (**B**) α4(+)α4(−), (**C**) α3(+)β2(−), (**D**) α3(+)α3(−) and (**E**) α7(+)α7(−). Predicted mutational energies are averages over 300 frames extracted from three molecular dynamics simulations. Standard deviations of these predictions are provided in Tables S1–S5.

FoldX is a well-established method used to predict mutational energies, and it was here employed to compute ΔΔGs for gGID*/nAChR complexes (Figure 6; Tables S1–S5). Since the four conserved cysteine residues and Pro-9 are crucial for maintaining the gGID* structure, ΔΔGs of these five positions were not calculated, except the P9A mutation as reported in the literature [20]. The ΔΔGs of 266 gGID* mutants were calculated in silico using rat α3β2 (two types of binding sites), α4β2 (two types of binding sites) and α7 nAChRs, and the predicted mutational energy changes are shown in Figure 6 and listed in Tables S1–S5. The mutational energy predictions suggest that the sequence of gGID* seems to be optimal for binding to the α4β2 nAChR because affinity could not be improved by a single amino-acid substitution apart from potentially position 4. Substitutions of Glu-4 into Phe, Tyr, Met, Lys or Arg amino acids are indeed predicted to increase affinity by about 1 kcal/mol. The side chain of this position is sandwiched between α4-E191, located in the C-loop, and β2-D171, located in the F-loop. A positively charged side chain Arg or Lys at position 4 would therefore establish attractive interactions with the negatively-charged side chains of D171 and E191 (Figure S4). We note that these interactions are highly solvated in the models, and we expect the stabilization to be decreased by interactions with water molecules and salt. These potentially stabilizing mutations at position 4 of gGID* are also suggested to increase affinity at all binding sites, and they would not contribute to the selectivity.

α-Conotoxins act by competitive inhibition with acetylcholine, and therefore, the strength of their affinity for nAChRs drives their inhibitory activity. We evaluated, using Matthews’ correlation coefficient (MCC), whether computed ΔΔGs could be used to predict a decrease in the activity (IC_50_ ratio) measured experimentally, as shown in Table 1. MCC gives a better estimate of the predictions than the accuracy (ACC) in our case because the number of substitutions leading to a decrease in activity/affinity (labeled “negative”) is different from the number of substitutions resulting in an increase or no change in activity/affinity (labeled “positive”) in each system. Optimal predictions of decreased activity were made if considering as significant loss an increase in IC_50_, corresponding to an at least 15 fold and a FoldX mutational energy of at least 2.3 kcal/mol. Among the two potential types of binding sites of the α4β2 nAChR, better predictions were made at the α4(+)β2(−) binding site than at the α4(+)α4(−) binding site according to MCC values, suggesting that gGID* inhibits α4β2 nAChR principally through interaction with the α4(+)β2(−) binding site. By contrast, the predictions suggest that the two types of binding sites of the α3β2 nAChR are similarly targeted. The best predictions were made at the α7 nAChR (90% accuracy and MCC of 0.67), which only has one type of binding site. FoldX models the conformational changes of side chains upon mutation but not of the main chain, and the impact of mutations at the interface with the flexible C-loop (Ala-10, Asn-14, Asn-15 and Val-18) were consequently less predictable.

We also investigated mutations that could potentially increase selectivity for the α4β2 nAChR compared to the α3β2 and α7 nAChRs. Supplementary Material Figure S5 provides differential mutational energies between α4(+)β2(−) and either α3(+)β2(−) or α7(+)α7(−). Considering a cut-off of at least 2.3 kcal/mol, several substitutions at position 10 into His, Ile, Arg, Trp and Tyr could potentially increase selectivity for α4β2 nAChR in regards to both α3β2 and α7 nAChRs. A10H, A10W and A10Y are predicted to be detrimental to the affinity for α4β2 nAChR (but even more so at the α3β2 and α7 nAChRs), and only A10I and A10R could preserve activity at α4β2 nAChR. As mentioned previously, the impact of the A10T substitution was wrongly predicted at the α4β2 nAChR to result in no decrease in activity, suggesting that the impact of the A10I and A10R substitutions might not be accurate. No other substitutions were predicted to improve selectivity at the α4β2 nAChR compared to the α3β2 nAChR. We also note that gGID is 20-fold more potent at the rat (128 nM) than at the human (3 μM) α4β2 nAChR [20,22], and this weak activity, along with the results of our analysis, suggests that it will be difficult to create a potent and selective variant of gGID. We therefore considered an alternative strategy to selectively inhibit the α4β2 nAChR and investigated the mutational energies of the ribbon isomer of GID, rGID. We have indeed shown that ribbon conotoxins can inhibit nAChRs, and in one case, ribbon α-conotoxin AuIB, with higher potency than the corresponding globular isomer [25,35].

#### 2.2.2. Full Sequence Amino Acid Scanning of rGID* at the Human α4(+)α4(−), α3(+)α3(−) and α7(+)α7(−) nAChR Binding Sites

**Figure 7:**
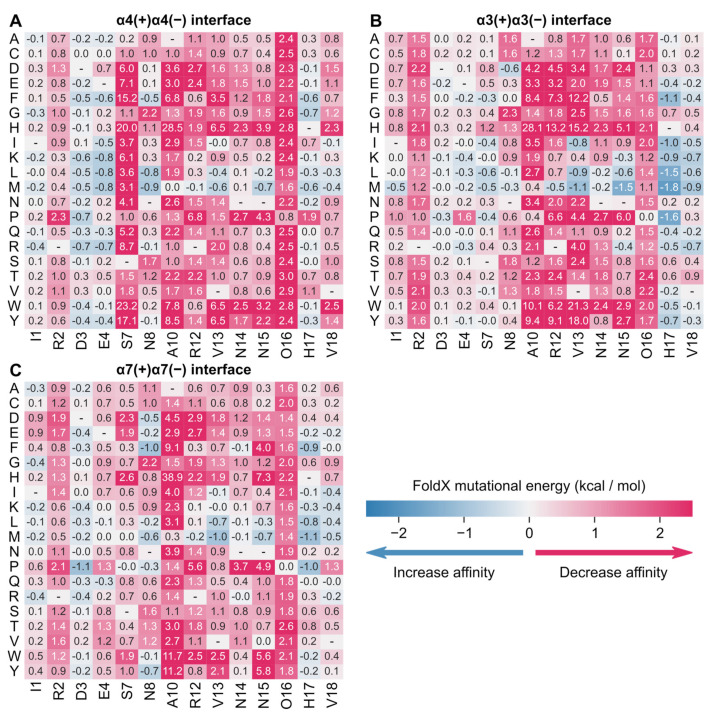
Mutational energies of rGID* predicted by FoldX in the context of three binding sites: (**A**) α4(+)α4(−), (**B**) α3(+)α3(−) and (**C**) α7(+)α7(−). Predicted mutational energies are averages over 300 frames extracted from three molecular dynamics simulations. Standard deviations of these predictions are provided in Tables S6–S8.

Using the models of the complex between rGID* and the human α4(+)α4(−), α3(+)α3(−) and α7(+)α7(−) nAChR binding sites, the ΔΔGs of a library of 285 rGID* mutants were calculated in silico (Figure 7; Tables S6–S8). No mutation at the α4(+)α4(−) binding site was predicted to increase affinity by more than 1 kcal/mol, but several substitutions at positions Asp-3, Glu-4, Asn-8, Val-13, Asn-15 and His-17 were predicted to have a minor stabilization effect, and their combination could further increase affinity. This information is valuable to help design a variant of rGID* with a high affinity for α4β2 nAChR. Substitutions at position 7 seem to be the most detrimental to affinity at α4(+)α4(−) (Figure 7), but the standard deviation of the predicted energies over three simulation replicates are of the same magnitude as the mean values (Table S7), indicating that predictions at this position are not reliable.

The difference in mutational energies of rGID at the α4(+)α4(−) and either α3(+)α3(−) or α7(+)α7(−) (Supplementary Material Figure S5) inform on the substitutions that are predicted to increase selectivity for α4β2 nAChR. Most substitutions at positions Ser-7, Hyp-17 and Val-18 substantially decreased the selectivity for α4β2 nAChR. By contrast, the three substitutions A10F, A10H and A10W increased selectivity for α4β2 nAChR by >2.3 kcal/mol compared to the two other nAChR subtypes, but at the expense of a substantial decrease in affinity by 20 kcal/mol at α4(+)α4(−). The substitution R12W increases selectivity for α4(+)α4(−) vs. α3(+)α3(−)/α7(+)α7(−) by 5.7/1.9 kcal/mol with only a minor 0.6 kcal/mol drop in affinity at the α4(+)α4(−) and therefore represents a better alternative to improve selectivity. Substitutions at positions 4 (E4F, E4I, E4K, E4L, E4M, E4R, E4Y), 8 (N8L) and 17 (H17G) are predicted to result in an increase in selectivity of 0.3–1.4 kcal/mol against both α3(+)α3(−)/α7(+)α7(−) receptors and an increase in affinity of 0.4–0.8 kcal/mol at the α4(+)α4(−) binding site. Alternatively, several substitutions of position 2 (R2D, R2E) increase selectivity at both receptors by 0.6–0.9 kcal/mol at the expense of a 0.8–1.3 kcal/mol decrease in affinity, representing a reasonable trade-off. These results suggest that rGID has potential for designing selective α4β2 nAChR inhibitors.

## 3. Discussion

In this study, we computationally investigated several variants of globular and ribbon GID* that could be designed to selectively and potently inhibit α4β2 nAChR. Seven models of GID* bound to α3β2 (two types of binding sites), α4β2 (two types of binding sites) and α7 nAChRs and three models of rGID* bound to α3β2, α4β2 and α7 nAChRs were built. The molecule models were used to calculate mutational energies. Some mutants of rGID* were identified as potential candidates for improving (α4)_3_(β2)_2_ nAChR stoichiometry inhibitors according to the ΔΔG results by targeting the α4-α4 binding site.

### 3.1. Quality of Molecular Models and Activity Predictions

The models were built using a crystal structure of human α4β2 nAChR as a template, but since the time we carried out these computations, several experimental structures of α3β4 and α7 nAChRs have been published [36,37] and could have alternatively been used as templates. Nevertheless, the binding sites of GID* peptides in our models overlay with the new structures with a backbone root-mean-square deviation between 1.2 and 1.7 Å, which is within the experimental resolution of these new structures, suggesting that our models are accurate. As further evidence of this accuracy, the models of gGID* in complex with the three nAChR subtypes provided reasonable explanations for the experimental mutational data. These data consist of IC_50_ activity measurements, which we considered as globally related to the affinity of GID* for nAChRs because GID* is a competitive inhibitor. Our analysis shows that a drop in activity by 15 fold can be reasonably well predicted apart for mutations that might result in change of conformations or reorientation of the peptide. Modeling of such changes of binding modes upon mutation can be accomplished by molecular dynamics simulations, and mutational energies can be predicted by methods such as MMPBSA [26,38]. Nevertheless, such computations are too time-consuming to be applied for the full amino acid scan of peptides.

### 3.2. The N- or C-Terminal Tail of the α-Conotoxin Can Modulate Activity on nAChRs

Most α-conotoxins do not bind to the α4β2 nAChR subtype [14,39]. A previous study found that the α4 (+)-R188 could prevent α-conotoxins to bind to α4β2 nAChRs [40]. Compared to other α-conotoxins, GID has a longer N-terminal tail. As described in the Results section, the α4 (+)-R188 interacts with the N-terminal tail of gGID* (Figure 2A), explaining the importance of this tail for gGID* activity at α4β2 nAChRs [20]. Additionally, Ala substitution or deletions in the tail significantly decreased activity at α3β2, α4β2 and α7 nAChRs [20]. These observations illustrate that the N-terminal tail is involved in modulating the activity of gGID* on α3β2, α4β2 and α7 nAChRs. Several other α-conotoxins also have an extended N- or C-terminal tail, including AnIB [19], LsIA [41] and Lo1a [42]. Like GID, the tail of these conotoxins was found to be involved in modulating the activity of these peptides [19,41,42]. For example, AnIB inhibited the α7 nAChR with IC_50_ of 76 nM, but deletion of a Gly in the N-terminal tail caused a loss of activity [19]. The truncation of the N-terminal tail of LsIA resulted in a drop ~10-fold in activity at α3β2 nAChR [41]. By contrast, truncation of the C-terminus of Lo1a results in an increase in activity by ~4000-fold at α7 nAChR [42]. Studying the mechanism of how modifications to N- or C- termini affect the activity of these peptides could help design more potent and selective nAChR antagonists.

### 3.3. Potential (α4)_3_(β2)_2_ nAChR Inhibitors Suggested by FoldX

The virtual screen of 266 gGID* point mutants did not reveal any novel peptides with large increased binding affinity for the α4β2 nAChR. This result is consistent with experimental mutational studies, which did not lead to a globular α-conotoxin GID* variant that would potently block α4β2 nAChR [20,21,22]. Alternative methods which could be used include mutating gGID* using non-natural amino acids, which has been used to increase the activity of α-conotoxin PnIA at α7 nAChR [43]. The design of ribbon α-conotoxins is an underexploited strategy with the potential to influence the inhibition of nAChRs [35].

The ΔΔG results for rGID* indicate that several substitutions at positions 3, 4, 8, 13, 15 and 17 could increase affinity for the human (α4)_3_(β2)_2_ nAChR. The effect of these individual substitutions are predicted to be small, but multiple substitutions could result in a high-affinity inhibitor. Our analysis also suggests that substitutions at positions 2, 4, 8, 10 and 17 of rGID could increase the selective inhibition of (α4)_3_(β2)_2_ nAChR compared to (α3)_3_(β2)_2_ and α7 nAChRs. We have identified some limitations to our predictions, especially when substitutions would impact the binding mode or conformation, and our results should be seen as a guide and not a replacement for experimental testing of activity.

## 4. Materials and Methods

### 4.1. Homology Modeling and Molecular Dynamics Simulation

Molecular models of the interaction between gGID* and the extracellular domain of rat α3β2, α4β2 and α7 nAChRs were built by homology using Modeller 9V18 [44] and the following crystal structures as templates: the complex between *Aplysia californica* acetylcholine-binding protein (AChBP) and conotoxin PnIA variant (PDB ID: 2BR8), and the human α4β2 nAChR (PDB ID: 5KXI; 6CNK; 6CNJ). The complexes between gGID* and the extracellular domain of (α3)_2_(β2)_3_, (α4)_2_(β2)_3_ and (α7)_5_ nAChR were modeled by assuming an interaction at the α3(+)β2(−), α3(+)α3(−), α4(+)β2(−), α4(+)α4(−) and α7(+)α7(−) orthosteric binding sites. The complex between rGID* and the extracellular domain of (α4)_3_(β2)_2_ nAChR was built by assuming an interaction at the α4(+)α4(−) orthosteric binding site using as template the molecular model of ribbon α-conotoxin AuIB in complex with the α3(+)α3(−) binding site of the α3β4 nAChR [25]. We similarly modeled the interaction of rGID* with the α3(+)α3(−) and the α7(+)α7(−) orthosteric binding sites. Our models previously suggested that the ribbon isomer of α-conotoxins adopt a shape that is more compatible with the α/α interface than the α/β interface. AuIB and GID also share the same cysteine framework and a similar first loop, which is critical for the ribbon isomer to bind to the nAChR according to our models. Modeler 9V18 was set up to create 100 models, and the best model was selected according to the lowest DOPE score [45].

The models were refined by a 50–100 ns molecular dynamics simulation in a solution of discrete water using the GROMACS 5.1.4 package [46] and the Amber99SB-ILDN protein force field [47]. Around 43,000 water molecules were added to each system, and the systems were neutralized by the addition of 20–40 sodium ions. Each system was first minimized with 10,000 steps using the steepest descent and was then gradually heated up from 50 to 300 K in the NVT ensemble over 200 ps. Position restraints imposed on the atoms were gradually removed from 1000 kJ mol^−1^ nm^−2^ to 0 kJ mol^−1^ nm^−2^ over 1 ns, followed by an unrestrained simulation until the last 20 ns of the simulations was stable according to the backbone root-mean-square deviation of the complex (up to 60 ns for gGID*/α4β2 complex, up to 100 ns for gGID*/α3β2, up to 70 ns for gGID*/α7 complex and 50 ns for rGID*/α4β2 complex). The stabilities of the conformation of the GID* peptides during the last 50 ns of the simulations are shown in Figure S6, where the backbone root-mean-square deviations are represented. All bonds involving hydrogen atoms were constrained with the LINCS algorithm [48], allowing the use of a 2 fs time step. The particle-mesh Ewald method [49] was used to compute long-range electrostatic interactions with a direct cutoff of 1.0 nm and a grid spacing of 0.12 nm. A 1.0 nm cutoff was used for van der Waals interactions. The Parrinello-Rahman barostat (time constant τ_P_ of 2.0 ps) and the V-rescale thermostat (time constant τ_T_ of 0.1 ps) were used to maintain the pressure and temperature at 1 atmosphere and 300K during the production runs. All systems were simulated in triplicate, and the last 20 ns were used for mutational energy prediction analysis.

### 4.2. Mutational Energy Calculation

For energy calculation, 100 frames were extracted at regular frequencies in the last 20 ns from each molecular dynamics simulation, and the resulting structures were optimized using FoldX 5.0 (http://foldx.crg.es/, accessed on 15 April 2021) *RepairPDB*. The *Buildmodel* command of FoldX was used to mutate the residues and build mutation models. The interaction energy and mutational energy of the complex between GID* and α3β2, α4β2 or α7 nAChR were calculated by the following equations: Δ*G*_binding_ = *G*_complex_ − (*G*_receptor_ + *G*_ligand_) and ΔΔ*G*_binding_ = Δ*G*_binding_ (mutant) − Δ*G*_binding_ (wildtype), which was conducted by the command *Positionscan*. The ΔΔG of each mutation is an automatic output in FoldX and generated in a separate file. For each system, the average ΔΔG of each mutation was computed as well as the standard deviations over the three simulations of each system. These average values are reported in Figure 6 and Figure 7 and the standard deviations are in the Supplementary Tables S1–S8.

### 4.3. Analysis of Energy Prediction Data

The experimental data and predicted ΔΔGs were divided into three classes. For experimental data: (i) decreased in activity (IC_50_ value of the mutant ≥ 15-fold from the IC_50_ value of the wildtype); (ii) similar activity (affinity value of the wildtype over affinity value of a mutant is within 15-fold); and (iii) improved in activity (15-fold improvement from the IC_50_ value of the wildtype). For the predicted ΔΔG, we binned the ΔΔG into three classes: (i) decreased in activity (ΔΔG ≥ 2.3 kcal/mol); (ii) similar activity (−2.3 kcal/mol < ΔΔG < 2.3 kcal/mol); and (iii) improved in activity (ΔΔG ≤ −2.3 kcal/mol).

A confusion matrix was used to analyze energy prediction data compared to experimental activity data. The terms of positive, negative, true and false were defined as follows: “positive” means that IC_50_ value decreased or showed no change in experimental data within 15-fold of IC_50_; “negative” means that IC_50_ value increased in experimental data more than 15-fold; “true” means that predicted mutational energies are consistent with experimental data; and “false” means that predicted mutational energies are not consistent with experimental data. The mutational energy results were classified as true positives (TP), false positives (FP), true negatives (TN) and false positives (FP). By these four values, the accuracy and Matthews’ correlation coefficient (MCC) were calculated using the equations as below:

$$\mathrm{Accuracy}\;=\;\frac{\mathrm{TP}\;+\;\mathrm{TN}}{\mathrm{TP}\;+\;\mathrm{TN}\;+\;\mathrm{FP}\;+\;\mathrm{FN}}\;\;\times \;100\%;\;\mathrm{MCC}=\;\frac{\mathrm{TP}\;\times \;\mathrm{TN}\;-\;\mathrm{FP}\;\times \;\mathrm{FN}\;}{\sqrt{\left(\mathrm{TP}\;+\;\mathrm{FP}\right)\left(\mathrm{TP}\;+\;\mathrm{FN}\right)\left(\mathrm{TN}\;+\;\mathrm{FP}\right)\left(\mathrm{TN}\;+\;\mathrm{FN}\right)}}$$

## 5. Conclusions

In summary, we have built several molecular models of the complexes between gGID* and several nAChR subtypes and determined that no single substitution is likely to increase affinity by more than 15 fold. The models also provide several important findings, including an explanation of the importance of the N-terminal tail of gGID* because of its interactions with the rat α4β2 nAChR. Finally, we modeled the complex between rGID* and the α/α interfaces of three nAChR subtypes and then used mutational energy predictions to suggest how rGID* could be modified to selectively and potently inhibit the (α4)3(β2)2 nAChR. This analysis suggested several substitutions of a ribbon conotoxin that could be combined to create potent and selective inhibitors of a single stoichiometry of a nAChR subtype. We anticipate that our results might inspire an increase in the use of ribbon isomers of α-conotoxins in studies aimed at designing nAChR ligands.

## Figures and Tables

**Table 1:** Comparison of experimental IC_50_ changes and predicted mutational energies (ΔΔG).

Substitution	α4β2 nAChR					α3β2 nAChR					α7 nAChR		
	IC_50_ Ratio ^a^	ΔΔG at the α4(+)β2(−)		ΔΔG at the α4(+)α4(−)		IC_50_ Ratio ^a^	ΔΔG at the α3(+)β2(−)		ΔΔG at the α3(+)α3(−)		IC_50_ Ratio ^a^	ΔΔG at the α7(+)α7(−)	
S7A	7.6	−0.2 ^b^	TP ^c^	0.0	TP	0.9	0.0	TP	0.2	TP	2.2	0.2	TP
N8A	>17	2.3	TN	1.8	FP	1.4	2.2	TP	1.8	TP	> 10	1.8	FP
P9A	>17	2.7	TN	2.9	TN	59	2.6	TN	2.8	TN	15	2.6	TN
A10S	0.8	0.7	TP	0.9	TP	6.4	1.0	TP	1.9	TP	-	-	-
A10T	>20	0.8	FP	2.5	TN	>2700	2.1	FP	3.2	TN	-	-	-
R12A	>17	1.8	FP	2.5	TN	3.6	2.2	TP	1.2	TP	8.3	1.5	TP
V13A	2.0	1.3	TP	1.5	TP	0.2	1.1	TP	2.3	TP	1.9	1.6	TP
V13F	>20	2.4	TN	8.3	TN	111	2.7	TN	10.5	TN	-	-	-
V13W	>20	6.7	TN	16.2	TN	>2700	8.2	TN	16.9	TN	-	-	-
V13L	>20	−0.5	FP	−0.7	FP	>2700	−0.7	FP	−0.2	FP	-	-	-
V13I	1.3	−0.3	TP	−0.3	TP	3.6	−0.5	TP	−0.3	TP	-	-	-
V13S	1.0	2.1	TP	1.9	TP	0.4	1.8	TP	2.3	FN	-	-	-
V13T	1.2	1.5	TP	1.1	TP	1.3	1.3	TP	1.4	TP	-	-	-
N14A	>17	1.5	FP	−0.2	FP	0.4	1.7	TP	1.9	TP	8.7	1.2	TP
N15A	>17	1.2	FP	1.1	FP	1.1	1.3	TP	0.8	TP	1.0	1.0	TP
N15K	>20	0.0	FP	−0.1	FP	>2700	0.2	FP	0.0	FP	-	-	-
N15H	>20	1.2	FP	2.0	FP	44	0.9	FP	3.4	TN	-	-	-
O16A	11	1.5	TP	2.4	FN	4.7	1.6	TP	1.2	TP	2.2	2.2	TP
H17A	11	0.4	TP	0.3	TP	0.9	0.2	TP	0.3	TP	0.8	0.1	TP
V18A	>17	1.4	FP	1.2	FP	14	1.0	TP	1.2	TP	1.3	1.2	TP
V18Y	>20	4.2	TN	1.8	FP	>2700	3.4	TN	1.0	FP	-	-	-
V18Q	>20	1.6	FP	0.7	FP	>2700	0.6	FP	0.7	FP	-	-	-
V18N	0.4	2.0	TP	1.1	TP	>2700	1.3	FP	1.0	FP	-	-	-
Accuracy		61%		57%			74%		74%			90%	
MCC ^d^		0.42		0.27			0.52		0.48			0.67	

^a^ IC_50_ values are from Millard et al. [20] and Banerjee et al. [21]. ^b^ Average ΔΔGs computed with FoldX over three replicate molecular dynamics simulations and 100 frames extracted from each simulation. The standard deviations of the mutational energies predicted over the three simulations are in Supplementary Material Tables S1–S8. ^c^ Classification of predictions as TP (True positive), TN (True negative), FP (False positive) and FN (False negative) by considering that IC_50_ ratios < 15 and predicted energies < 2.3 kcal/mol are labeled “positive” and otherwise labeled “negative”. ^d^ Matthews’ correlation coefficient (1.00 is perfect prediction, 0.00 is random prediction).

## Data Availability

Original data and Excel files are available on request.

## Supplementary Materials

The following are available online at https://www.mdpi.com/article/10.3390/md19090482/s1, Figure S1: Overlay of the binding sites of gGID* and rGID*, Figure S2: Conformational exploration of the N-terminal tail of gGID*, Figure S3: Molecular models of the complexes gGID*/α3(+)α3(−), gGID*/α4(+)α4(−), rGID*/α3(+)α3(−), and rGID*/α7(+)α7(−), Figure S4: Molecular models of the E4K and E4R variants of gGID* in the context of the α4(+)β2(−) binding site, Figure S5: Differential mutational energies predicted by FoldX, Figure S6: Backbone root-mean-square deviations (RMSD) of gGID* during the last 50 ns of molecular dynamics simulations, Figure S7: Backbone root-mean-square deviations (RMSD) of gGID* during the last 50 ns of molecular dynamics simulations, Table S1: Mutational energies of gGID* at α3(+)α3(−) interface as predicted by FoldX 5.0, Table S2: Mutational energies of gGID* at α3(+)β2(−) interface as predicted by FoldX 5.0, Table S3: Mutational energies of gGID* at α4(+)α4(−) interface as predicted by FoldX 5.0, Table S4: Mutational energies of gGID* at α4(+)β2(−) interface as predicted by FoldX 5.0, Table S5: Mutational energies of gGID* at α7(+)α7(−) interface as predicted by FoldX 5.0, Table S6: Mutational energies of rGID* at α3(+)α3(−) interface as predicted by FoldX 5.0, Table S7: Mutational energies of rGID* at α4(+)α4(−) interface as predicted by FoldX 5.0; Table S8: Mutational energies of rGID* at α7(+)α7(−) interface as predicted by FoldX 5.0.
