# Supplementary material for: Interactions of Globular and Ribbon [γ4E]GID with α4β2 Neuronal Nicotinic Acetylcholine Receptor
Source: Mar Drugs. 2021 Aug 26;19(9):482. doi: 10.3390/md19090482 (PMC8469569; doi:10.3390/md19090482)
Supplement: Supplementary file 1 [file marinedrugs-19-00482-s001.zip › marinedrugs-1204582-supplementary.pdf]

Supplementary Materials

Interactions of Globular and Ribbon [γ4E]GID with α4β2 Neuronal Nicotinic Acetylcholine Receptor

Xiaosa Wu [1,2], David J. Craik [1,*] and Quentin Kaas [1,*]

[1] Institute for Molecular Bioscience, Australian Research Council Centre of Excellence for Innovations in Peptide and Protein Science, The University of Queensland, Brisbane, Queensland 4072, Australia; xiaosa.wu@uq.net.au

[2] National Institutes of Health, Building 35A, Room 3D-953B, 35 Convent Drive, Bethesda, MD 20892-3701

* Correspondence: d.craik@imb.uq.edu.au (D.J.C.); q.kaas@imb.uq.edu.au (Q.K.).

List of Figures

Page 2 **Figure S1**: Overlay of the binding sites of gGID* and rGID*.
Page 3 **Figure S2**: Conformational exploration of the N-terminal tail of gGID*
Page 4 **Figure S3**: Molecular models of the complexes gGID*/α3(+)α3(−), gGID*/α4(+)α4(−), rGID*/α3(+)α3(−), and rGID*/α7(+)α7(−).
Page 5 **Figure S4**: Molecular models of the E4K and E4R variants of gGID* in the context of the α4(+)β2(−) binding site.
Page 6 **Figure S5**: Differential mutational energies predicted by FoldX.
Page 7 **Figure S6**: Backbone root-mean-square deviations (RMSD) of gGID* during the last 50 ns of molecular dynamics simulations.
Page 8 **Figure S7**: Backbone root-mean-square deviations (RMSD) of gGID* during the last 50 ns of molecular dynamics simulations.

List of Tables

Page 9 **Table S1**: Mutational energies of gGID* at α3(+)α3(−) interface as predicted by FoldX 5.0.
Page 10 **Table S2**: Mutational energies of gGID* at α3(+)β2(−) interface as predicted by FoldX 5.0.
Page 11 **Table S3**: Mutational energies of gGID* at α4(+)α4(−) interface as predicted by FoldX 5.0.
Page 12 **Table S4**: Mutational energies of gGID* at α4(+)β2(−) interface as predicted by FoldX 5.0.
Page 13 **Table S5**: Mutational energies of gGID* at α7(+)α7(−) interface as predicted by FoldX 5.0.
Page 14 **Table S6**: Mutational energies of rGID* at α3(+)α3(−) interface as predicted by FoldX 5.0.
Page 15 **Table S7**: Mutational energies of rGID* at α4(+)α4(−) interface as predicted by FoldX 5.0.
Page 16 **Table S8**: Mutational energies of rGID* at α7(+)α7(−) interface as predicted by FoldX 5.0.

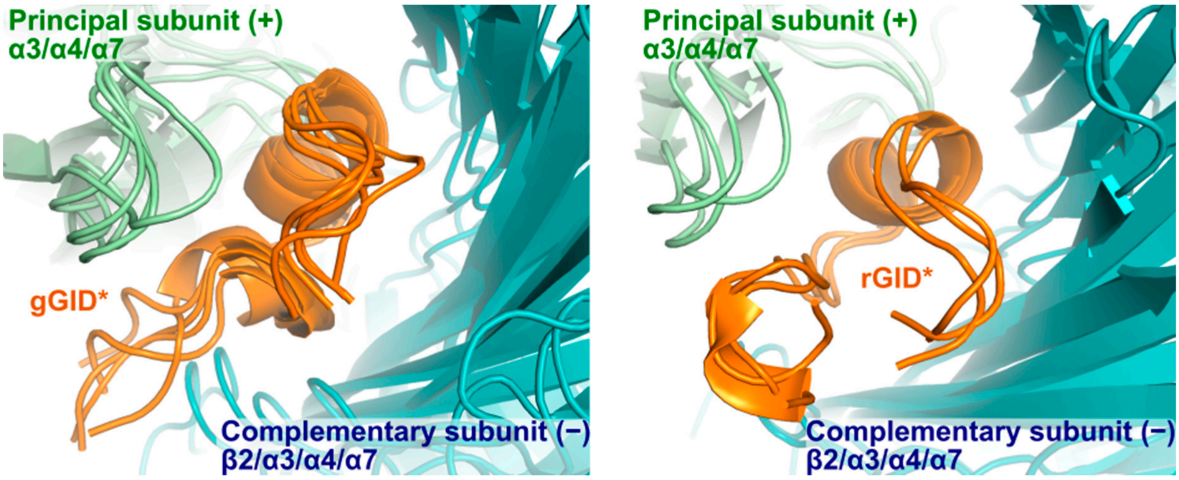

Figure S1: Overlay of the binding sites of gGID* (left) in the last frames of the simulations of the complexes α3(+)β2(−)/gGID*, α3(+)α3(−)/gGID*, α4(+)β2(−)/gGID*, α4(+)α4(−)/gGID* and α7(+)α7(−)/gGID*, and of the binding sites of rGID* (right) in the last frames of the simulations of α3(+)α3(−)/rGID*, α4(+)α4(−)/gGID* and α7(+)α7(−)/gGID*. The principal subunits are in green, GID* peptides in orange and the complementary subunit in blue. The simulations suggest a similar binding mode of gGID* and rGID* in all binding sites.

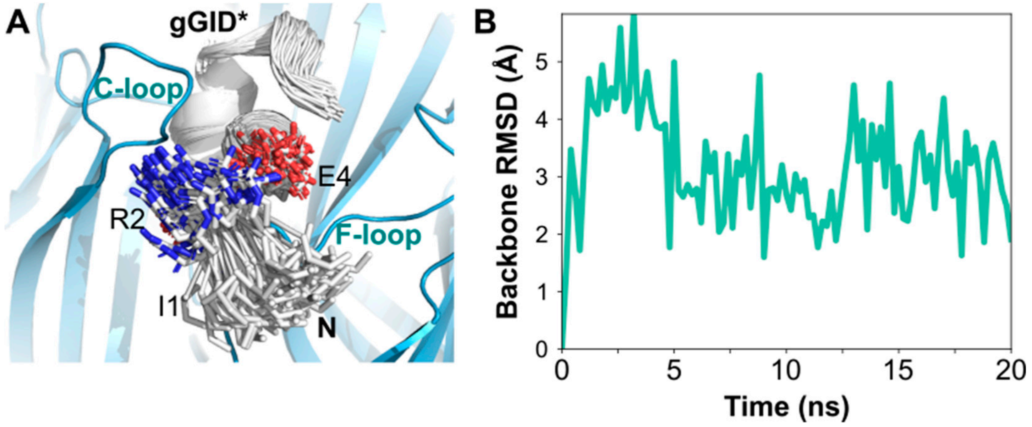

Figure S2: Conformational exploration of the N-terminal tail of gGID* during the last 20 ns of a molecular dynamics simulation of gGID*/α4β2 nAChR. (**A**) Overlay of regularly extracted 100 frames during the last 20 ns of the simulation that were used for the FoldX analysis. The four-residues (IRDE) at the N-terminus of gGID* underwent larger conformational fluctuation than other part of the peptide, albeit these conformations are restrained to a space defined between the C-loop and the F-loop of the binding site. (**B**) Backbone root-mean-square fluctuations of the backbone atoms of the first four residues of gGID* after overlay of the α-helix backbone atoms.

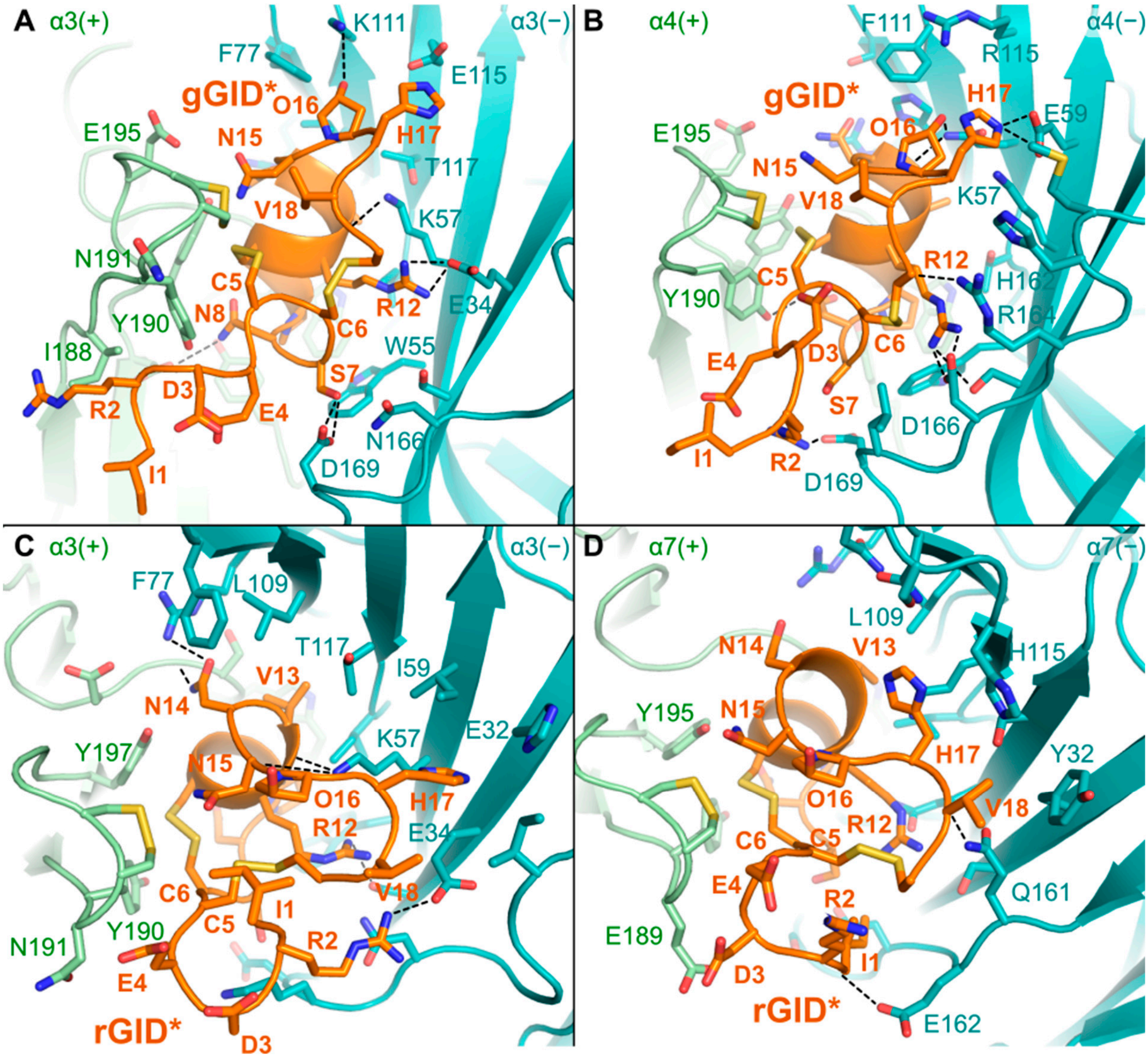

Figure S3: Molecular models of the complexes (**A**) gGID*/α3(+)α3(−), (**B**) gGID*/α4(+)α4(−), (**C**) rGID*/α3(+)α3(−), and (**D**) rGID*/α7(+)α7(−). The GID* peptides are in orange, the principal subunits in green and complementary subunits in blue. The represented models are the last frame of the molecular dynamics simulations used to refine each system.

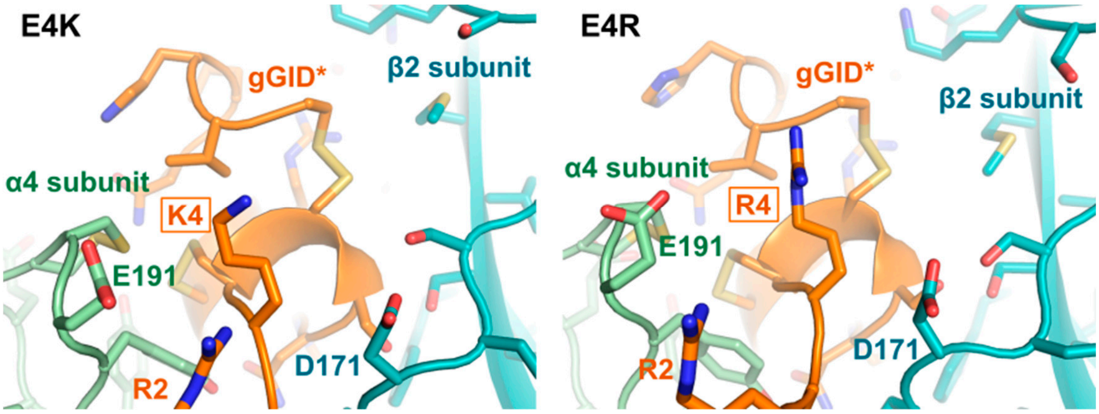

Figure S4: Molecular models of the E4K (left) and E4R (right) variants of gGID* in the context of the α4(+)β2(−) binding site. The molecular models were generated by FoldX using a frame from the molecular dynamics simulation of gGID*/α4β2 nAChR. The basic side chains of K4 and R4 are sandwiched by the negative charge-bearing side chains of D171 and E191 from the β2 and α4 subunits, respectively.

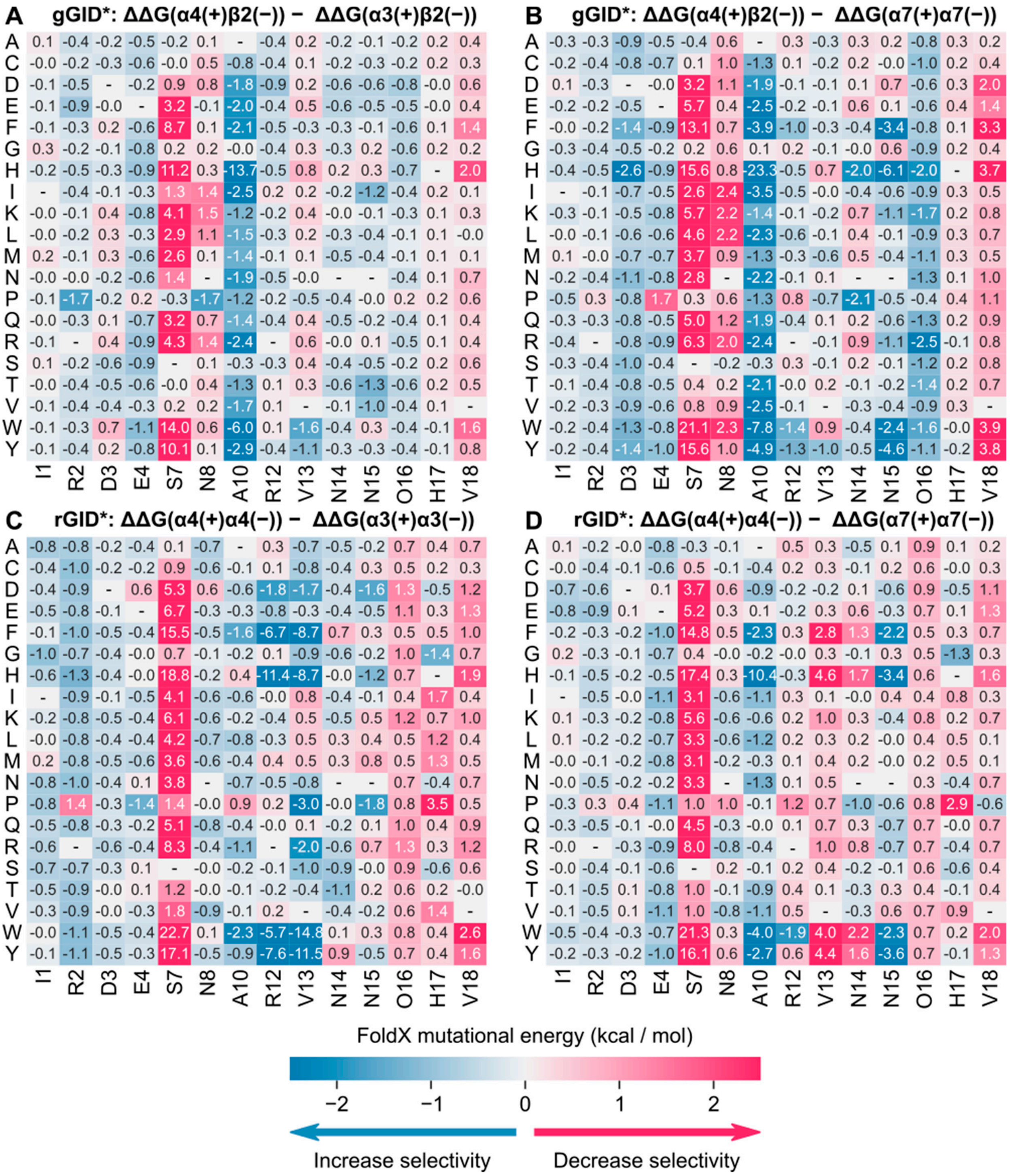

Figure S5: Differential mutational energies predicted by FoldX: (**A**) between the gGID*/α4(+)β2(−) and the gGID*/α3(+)β2(−) systems, (**B**) between the gGID*/α4(+)β2(−) and the gGID*/α7(+)α7(−) systems, (**C**) between the rGID*/α4(+)α4(−) and the rGID*/α3(+)α3(−) systems, and (**D**) between the rGID*/α4(+)α4(−) and the rGID*/α7(+)α7(−) systems.

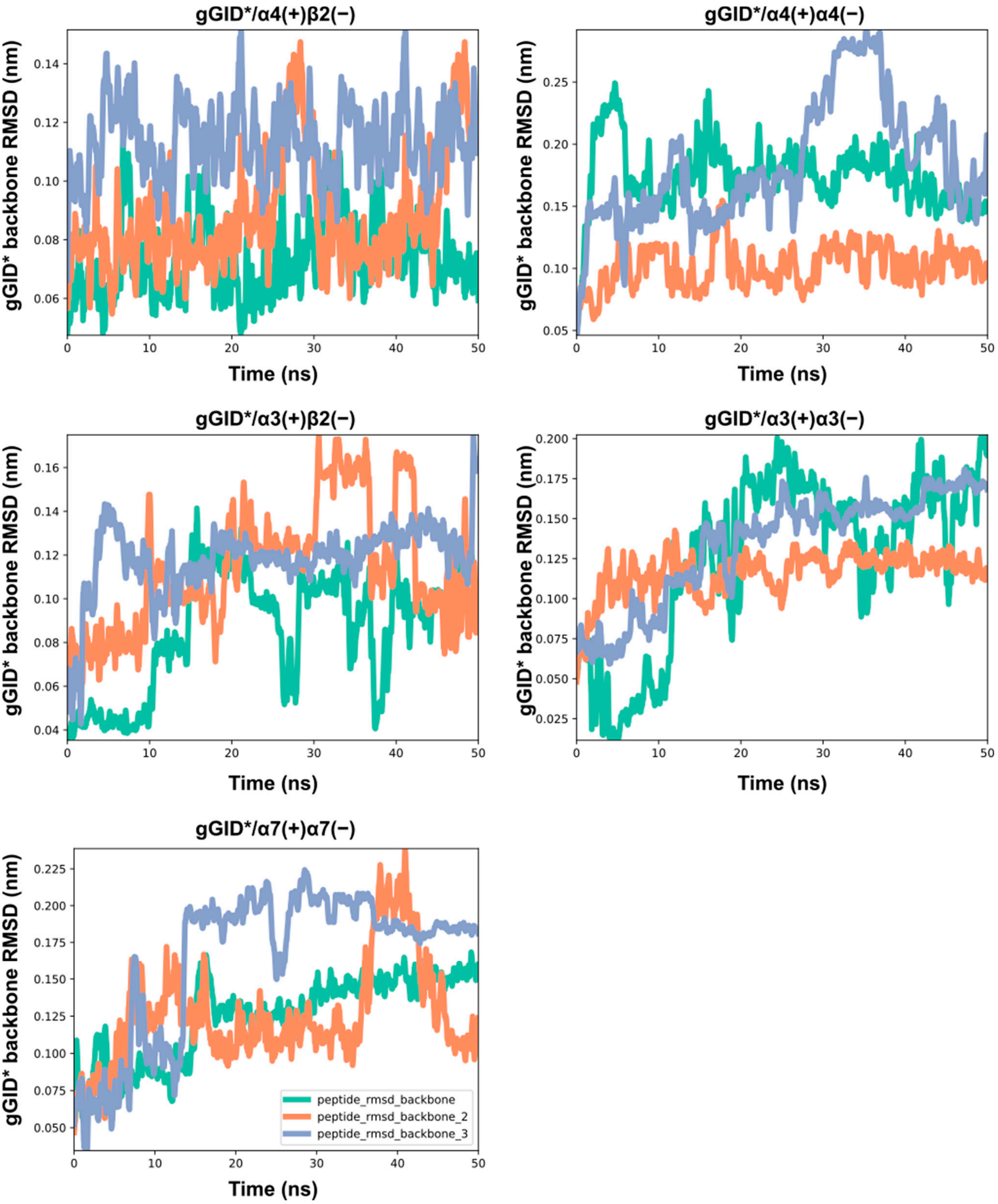

Figure S6: Backbone root-mean-square deviations (RMSD) of gGID* from its conformation at 0 ns from the last 50 ns of the molecular dynamics simulations of gGID* in complex with five

binding sites. The backbone RMSD traces are shown in different colors for the three simulations carried out for each system.

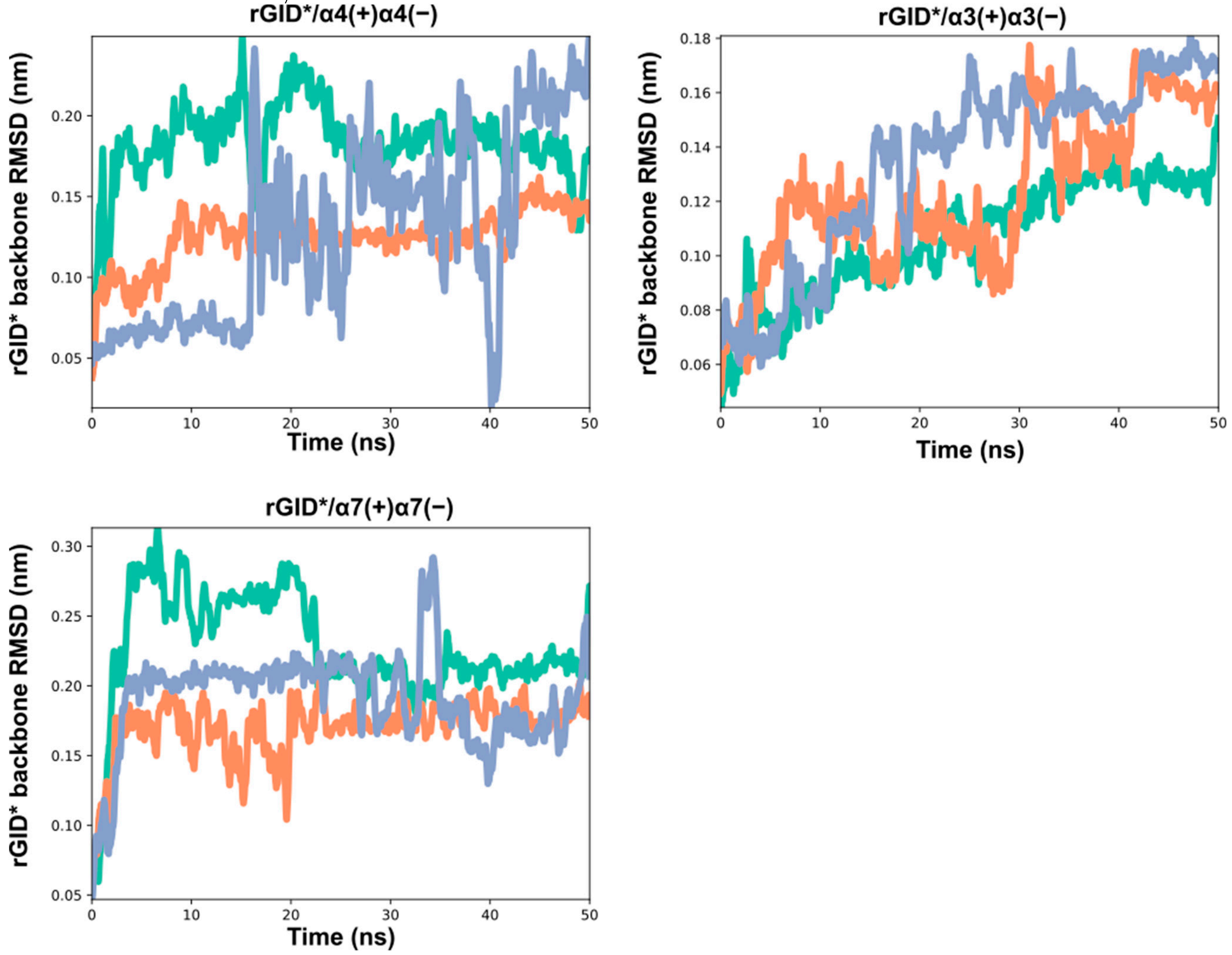

Figure S7: Backbone root-mean-square deviations (RMSD) of rGID* from its conformation at 0 ns from the last 50 ns of the molecular dynamics simulations of rGID* in complex with three binding sites. The backbone RMSD traces are shown in different colors for the three simulations carried out for each system.

Table S1: Mutational energies (kcal/mol) of gGID* at $\alpha3(+)\alpha3(-)$ interface as predicted by FoldX 5.0.

AA	Ile-1	Arg-2	Asp-3	Glu-4	Ser-7	Asn-8	Ala-10	Arg-12	Val-13	Asn-14	Asn-15	Hyp-16	His-17	Val-18
**A**	−0.1 ± 0.6[a]	0.9 ± 0.1	0.3 ± 0.3	0.2 ± 0.9	0.2 ± 0.1	1.8 ± 0.1		1.2 ± 0.3	2.3 ± 0.1	1.9 ± 0.4	0.8 ± 0.3	1.2 ± 0.1	0.3 ± 0.1	1.2 ± 0.2
**C**	0.1 ± 0.3	0.8 ± 0.3	0.2 ± 0.3	0.7 ± 0.8	0.1 ± 0.0	1.1 ± 0.0	2.0 ± 0.4	1.6 ± 0.2	1.9 ± 0.1	2.0 ± 0.4	0.5 ± 0.3	1.4 ± 0.1	0.1 ± 0.3	0.8 ± 0.2
**D**	0.3 ± 0.8	1.2 ± 0.3		1.1 ± 1.1	2.0 ± 0.7	0.1 ± 0.4	5.1 ± 0.6	2.6 ± 0.3	2.8 ± 0.3	2.3 ± 0.1	2.8 ± 0.2	0.9 ± 0.2	−0.0 ± 0.2	2.0 ± 0.7
**E**	0.3 ± 0.5	0.8 ± 0.2	−0.0 ± 0.2		1.4 ± 0.6	0.7 ± 0.5	4.2 ± 1.3	2.8 ± 0.3	2.6 ± 0.1	2.7 ± 0.5	2.4 ± 0.5	1.0 ± 0.1	−0.1 ± 0.1	1.4 ± 0.5
**F**	−0.1 ± 0.3	0.3 ± 0.2	−0.3 ± 0.5	0.3 ± 0.8	1.3 ± 0.4	−0.7 ± 0.4	13.2 ± 3.3	−0.5 ± 0.5	10.5 ± 1.5	1.1 ± 0.7	1.7 ± 2.1	1.0 ± 0.1	−0.1 ± 0.2	0.6 ± 0.7
**G**	−0.2 ± 0.7	0.8 ± 0.4	0.4 ± 0.1	0.5 ± 1.2	0.4 ± 0.3	2.8 ± 0.1	0.8 ± 0.2	2.4 ± 0.2	3.1 ± 0.0	2.8 ± 0.6	1.8 ± 0.6	1.2 ± 0.2	−0.1 ± 0.6	1.5 ± 0.4
**H**	0.3 ± 0.4	0.8 ± 0.2	0.4 ± 0.2	1.0 ± 1.0	2.6 ± 1.3	1.1 ± 0.7	39.5 ± 2.2	2.1 ± 0.4	11.2 ± 2.7	2.3 ± 0.6	3.4 ± 3.3	1.6 ± 0.1		1.6 ± 0.8
**I**		0.8 ± 0.1	−0.0 ± 0.6	0.6 ± 0.2	0.2 ± 0.3	1.0 ± 0.3	5.8 ± 1.0	2.1 ± 0.3	−0.3 ± 0.0	1.9 ± 0.3	0.3 ± 0.9	1.5 ± 0.3	1.4 ± 0.9	−0.0 ± 0.2
**K**	−0.3 ± 0.1	0.1 ± 0.1	−0.5 ± 0.3	−0.1 ± 0.7	−0.2 ± 0.3	0.4 ± 0.2	3.8 ± 0.7	−0.0 ± 0.6	0.5 ± 0.2	1.6 ± 0.6	−0.0 ± 0.7	1.0 ± 0.3	−0.2 ± 0.3	0.1 ± 0.2
**L**	−0.1 ± 0.1	0.4 ± 0.0	−0.5 ± 0.5	0.1 ± 0.7	−0.1 ± 0.6	0.0 ± 0.3	4.4 ± 0.7	−0.3 ± 0.4	−0.2 ± 0.1	0.7 ± 0.4	−0.0 ± 0.5	1.1 ± 0.3	0.1 ± 0.1	0.3 ± 0.1
**M**	−0.4 ± 0.1	0.3 ± 0.1	−0.4 ± 0.5	−0.2 ± 0.5	−0.7 ± 0.1	−0.2 ± 0.2	2.3 ± 0.7	−0.8 ± 0.5	−0.4 ± 0.1	0.8 ± 0.5	−0.4 ± 0.6	0.9 ± 0.3	0.1 ± 0.1	0.1 ± 0.2
**N**	0.1 ± 0.5	0.6 ± 0.3	−0.0 ± 0.2	0.3 ± 0.9	0.5 ± 0.4		4.8 ± 0.6	1.5 ± 0.2	2.3 ± 0.1			1.3 ± 0.1	−0.1 ± 0.4	1.0 ± 0.5
**P**	0.5 ± 0.4	2.3 ± 0.2	1.0 ± 0.5	−0.2 ± 0.3	0.8 ± 0.5	5.2 ± 0.7	0.3 ± 0.4	8.2 ± 0.7	4.0 ± 0.3	5.9 ± 0.4	3.8 ± 0.6	0.1 ± 0.2	1.6 ± 1.8	2.1 ± 0.6
**Q**	−0.0 ± 0.3	0.5 ± 0.2	−0.1 ± 0.2	0.0 ± 0.6	0.2 ± 0.3	0.8 ± 0.3	4.4 ± 0.9	1.5 ± 0.3	1.2 ± 0.1	1.1 ± 0.5	0.7 ± 0.4	1.2 ± 0.2	−0.0 ± 0.3	0.7 ± 0.4
**R**	−0.5 ± 0.2		−0.3 ± 0.2	−0.0 ± 1.0	−0.0 ± 0.5	−0.5 ± 0.4	3.3 ± 0.8		1.2 ± 0.7	2.0 ± 0.7	−0.6 ± 0.5	0.9 ± 0.5	−0.1 ± 0.3	0.1 ± 0.3
**S**	0.0 ± 0.8	0.8 ± 0.4	0.4 ± 0.1	0.7 ± 1.1		1.9 ± 0.1	1.0 ± 0.2	1.6 ± 0.3	2.3 ± 0.0	2.5 ± 0.5	1.1 ± 0.3	1.2 ± 0.1	0.1 ± 0.5	1.4 ± 0.3
**T**	−0.0 ± 0.4	1.2 ± 0.5	0.6 ± 0.2	1.6 ± 1.0	0.4 ± 0.3	1.1 ± 0.1	3.2 ± 0.3	2.9 ± 0.1	1.4 ± 0.0	2.7 ± 0.3	0.9 ± 0.2	1.7 ± 0.1	0.7 ± 0.1	1.3 ± 0.3
**V**	0.2 ± 0.2	1.1 ± 0.2	0.2 ± 0.5	1.0 ± 0.3	0.5 ± 0.3	1.2 ± 0.2	4.1 ± 0.7	2.4 ± 0.2		2.1 ± 0.2	0.2 ± 0.6	1.7 ± 0.2	1.1 ± 0.5	
**W**	−0.1 ± 0.2	0.8 ± 0.1	0.2 ± 0.4	0.9 ± 0.9	2.1 ± 1.1	0.2 ± 0.5	20.6 ± 3.0	1.8 ± 0.5	16.9 ± 0.9	2.7 ± 0.5	2.8 ± 2.4	1.3 ± 0.1	0.3 ± 0.1	1.6 ± 0.9
**Y**	−0.1 ± 0.1	0.4 ± 0.2	−0.3 ± 0.4	0.6 ± 0.7	1.4 ± 0.4	−0.6 ± 0.5	13.7 ± 2.1	−0.1 ± 0.6	16.6 ± 1.9	1.7 ± 0.6	3.1 ± 3.2	1.1 ± 0.1	−0.2 ± 0.1	1.0 ± 0.9

[a] Standard deviation computed from three replicate simulations and mutational energies predicted from 100 frames extracted from the last 20 ns from each simulation.

Table S2: Mutational energies (kcal/mol) of gGID* at $\alpha3(+)\beta2(-)$ interface as predicted by FoldX 5.0.

AA	Ile-1	Arg-2	Asp-3	Glu-4	Ser-7	Asn-8	Ala-10	Arg-12	Val-13	Asn-14	Asn-15	Hyp-16	His-17	Val-18
**A**	−0.1 ± 0.2[a]	1.2 ± 0.2	−0.1 ± 0.1	0.1 ± 0.8	−0.0 ± 0.1	2.2 ± 0.2		2.2 ± 0.3	1.1 ± 0.1	1.7 ± 0.5	1.3 ± 0.3	1.6 ± 0.1	0.2 ± 0.1	1.0 ± 0.3
**C**	−0.1 ± 0.2	1.1 ± 0.2	0.1 ± 0.2	0.1 ± 0.8	0.5 ± 0.2	1.7 ± 0.5	1.3 ± 0.6	2.4 ± 0.3	1.2 ± 0.1	1.4 ± 0.6	1.4 ± 0.3	1.7 ± 0.2	0.1 ± 0.2	0.8 ± 0.4
**D**	0.3 ± 0.1	1.9 ± 0.2		0.3 ± 0.3	4.5 ± 1.0	−0.1 ± 1.0	4.7 ± 1.1	4.8 ± 0.1	2.3 ± 0.3	3.3 ± 0.2	3.8 ± 0.4	2.9 ± 0.3	0.4 ± 0.0	3.0 ± 1.2
**E**	0.1 ± 0.1	1.8 ± 0.1	−0.3 ± 0.1		4.3 ± 2.2	0.7 ± 0.8	3.0 ± 1.6	3.7 ± 0.3	1.8 ± 0.2	2.8 ± 0.9	3.0 ± 0.3	2.2 ± 0.2	0.1 ± 0.1	2.0 ± 0.8
**F**	0.2 ± 0.1	1.0 ± 0.2	−0.6 ± 0.1	−0.7 ± 0.9	4.9 ± 3.3	0.2 ± 0.6	9.1 ± 3.5	0.9 ± 0.3	2.7 ± 0.4	1.6 ± 0.8	1.0 ± 0.3	2.0 ± 0.5	−0.8 ± 0.4	2.2 ± 1.9
**G**	−0.3 ± 0.3	1.2 ± 0.1	0.3 ± 0.1	0.8 ± 0.6	0.5 ± 0.1	2.9 ± 0.3	1.4 ± 0.1	3.4 ± 0.2	1.9 ± 0.0	2.1 ± 0.5	2.6 ± 0.3	2.1 ± 0.2	0.4 ± 0.1	1.8 ± 0.7
**H**	0.0 ± 0.3	1.1 ± 0.1	0.0 ± 0.2	0.2 ± 0.6	5.7 ± 2.9	1.3 ± 0.7	30.3 ± 11.7	2.6 ± 0.0	4.4 ± 2.1	2.2 ± 1.0	0.9 ± 0.1	2.8 ± 0.7		3.0 ± 1.4
**I**		1.3 ± 0.2	−0.1 ± 0.2	−0.2 ± 0.6	2.3 ± 0.8	2.0 ± 0.3	3.4 ± 2.4	1.8 ± 0.6	−0.5 ± 0.1	1.8 ± 0.8	2.2 ± 0.1	2.0 ± 0.5	−0.2 ± 0.2	0.3 ± 0.2
**K**	−0.4 ± 0.1	0.5 ± 0.2	−0.7 ± 0.1	−0.5 ± 0.8	1.6 ± 2.0	1.2 ± 0.7	2.1 ± 2.0	1.1 ± 0.3	0.4 ± 0.1	1.4 ± 0.5	0.1 ± 0.5	1.4 ± 0.3	−0.6 ± 0.1	0.6 ± 0.6
**L**	−0.1 ± 0.0	1.0 ± 0.2	−0.7 ± 0.1	−0.5 ± 0.9	1.9 ± 1.5	1.1 ± 1.0	2.6 ± 2.0	0.3 ± 0.2	−0.7 ± 0.3	0.8 ± 0.9	0.9 ± 0.2	1.1 ± 0.2	−0.4 ± 0.2	0.6 ± 0.5
**M**	−0.4 ± 0.0	0.9 ± 0.3	−0.9 ± 0.0	−0.5 ± 0.9	1.0 ± 1.2	0.6 ± 0.6	0.7 ± 1.4	0.1 ± 0.2	−1.0 ± 0.1	1.0 ± 0.8	0.6 ± 0.5	0.6 ± 0.2	−0.5 ± 0.3	0.3 ± 0.4
**N**	−0.1 ± 0.3	0.9 ± 0.2	−0.2 ± 0.1	−0.3 ± 0.7	2.1 ± 0.9		3.9 ± 1.4	2.9 ± 0.5	1.8 ± 0.1			1.7 ± 0.2	0.1 ± 0.0	1.3 ± 0.7
**P**	0.1 ± 0.2	3.4 ± 1.4	−0.6 ± 0.1	2.0 ± 1.2	1.5 ± 0.3	6.8 ± 1.1	1.3 ± 0.6	8.6 ± 1.1	3.0 ± 0.8	3.4 ± 0.8	5.2 ± 0.3	0.0 ± 0.2	1.4 ± 0.3	3.1 ± 0.9
**Q**	−0.2 ± 0.1	0.9 ± 0.1	−0.4 ± 0.1	−0.2 ± 0.6	2.3 ± 1.6	1.5 ± 0.2	2.3 ± 1.7	2.0 ± 0.1	1.1 ± 0.1	1.4 ± 0.6	1.2 ± 0.2	1.6 ± 0.1	−0.2 ± 0.1	1.2 ± 0.6
**R**	−0.7 ± 0.2		−0.8 ± 0.1	−0.5 ± 0.7	1.8 ± 1.8	1.1 ± 0.3	2.7 ± 3.3		1.6 ± 0.4	1.6 ± 0.7	−0.2 ± 0.5	1.3 ± 0.3	−0.6 ± 0.1	0.6 ± 0.7
**S**	−0.1 ± 0.3	0.9 ± 0.1	0.2 ± 0.1	0.5 ± 0.8		1.7 ± 0.1	0.9 ± 0.1	2.6 ± 0.3	1.8 ± 0.1	2.0 ± 0.4	2.3 ± 0.3	1.6 ± 0.2	0.2 ± 0.1	1.5 ± 0.6
**T**	0.0 ± 0.2	1.2 ± 0.3	0.6 ± 0.1	0.4 ± 0.7	1.0 ± 0.4	1.2 ± 0.3	2.1 ± 1.1	2.7 ± 0.3	1.3 ± 0.0	2.4 ± 0.6	3.4 ± 0.3	2.4 ± 0.2	0.4 ± 0.1	1.2 ± 0.2
**V**	0.2 ± 0.1	1.4 ± 0.3	0.2 ± 0.2	−0.1 ± 0.8	1.6 ± 0.4	1.9 ± 0.1	2.2 ± 1.5	2.1 ± 0.5		1.9 ± 0.6	2.3 ± 0.1	2.4 ± 0.3	0.1 ± 0.1	
**W**	−0.1 ± 0.1	1.2 ± 0.3	−0.5 ± 0.1	0.3 ± 0.6	8.3 ± 6.2	2.2 ± 0.4	13.7 ± 6.8	1.4 ± 1.1	8.2 ± 1.5	3.1 ± 0.5	1.5 ± 0.2	1.9 ± 0.8	−0.4 ± 0.2	3.0 ± 1.5
**Y**	0.2 ± 0.1	1.1 ± 0.2	−0.5 ± 0.1	−0.5 ± 0.7	6.0 ± 3.9	0.8 ± 0.7	10.4 ± 5.1	1.3 ± 0.6	5.3 ± 0.9	2.1 ± 0.6	1.5 ± 0.3	2.1 ± 0.6	−0.8 ± 0.3	3.4 ± 3.0

[a] Standard deviation computed from three replicate simulations and mutational energies predicted from 100 frames extracted from the last 20 ns from each simulation.

Table S3: Mutational energies (kcal/mol) of gGID* at α4(+)α4(−) interface as predicted by FoldX 5.0.

AA	I1	R2	D3	E4	S7	N8	A10	R12	V13	N14	N15	O16	H17	V18
**A**	−0.1 ± 0.4[a]	1.2 ± 0.7	0.1 ± 1.0	0.4 ± 0.3	0.0 ± 0.3	1.8 ± 0.6		2.5 ± 1.1	1.5 ± 0.2	−0.2 ± 0.2	1.1 ± 0.7	2.4 ± 0.6	0.2 ± 0.1	1.2 ± 0.5
**C**	0.0 ± 0.2	1.1 ± 0.6	−0.0 ± 1.0	0.7 ± 0.4	0.5 ± 0.6	1.3 ± 0.2	1.5 ± 0.8	2.6 ± 1.2	1.2 ± 0.1	0.1 ± 0.2	0.7 ± 0.7	2.5 ± 0.5	−0.0 ± 0.2	0.9 ± 0.6
**D**	0.2 ± 0.2	1.7 ± 1.0		0.7 ± 0.3	3.6 ± 1.5	−0.4 ± 0.7	4.5 ± 1.6	4.0 ± 0.8	2.2 ± 0.4	1.4 ± 0.5	2.1 ± 0.7	2.1 ± 0.3	−0.2 ± 0.2	2.1 ± 1.4
**E**	0.1 ± 0.2	1.3 ± 0.8	0.0 ± 0.6		2.7 ± 1.7	0.1 ± 0.9	3.1 ± 2.1	3.7 ± 0.8	2.1 ± 0.5	1.6 ± 0.4	1.8 ± 1.1	1.8 ± 0.4	−0.2 ± 0.2	1.4 ± 1.2
**F**	0.0 ± 0.4	0.6 ± 0.9	−0.6 ± 0.7	0.3 ± 0.3	4.9 ± 3.5	−0.4 ± 1.0	11.7 ± 4.8	3.1 ± 1.2	8.3 ± 3.6	0.9 ± 0.6	0.4 ± 1.1	2.3 ± 0.5	−0.1 ± 0.1	1.1 ± 1.7
**G**	−0.2 ± 0.6	1.3 ± 0.6	0.1 ± 1.6	1.0 ± 0.3	0.6 ± 0.0	2.9 ± 0.5	0.9 ± 0.2	3.5 ± 1.1	2.3 ± 0.4	0.6 ± 0.3	2.4 ± 0.9	2.4 ± 0.6	−0.5 ± 0.2	1.5 ± 0.8
**H**	−0.1 ± 0.5	1.1 ± 0.7	0.0 ± 0.8	1.0 ± 0.2	8.7 ± 6.4	1.2 ± 1.0	33.8 ± 7.6	5.3 ± 1.7	14.1 ± 5.0	1.4 ± 0.3	2.0 ± 1.5	3.0 ± 0.5		2.0 ± 2.1
**I**		0.9 ± 0.7	0.3 ± 0.7	0.5 ± 0.7	1.3 ± 1.9	1.5 ± 0.5	4.5 ± 2.0	2.1 ± 0.7	−0.3 ± 0.2	0.6 ± 0.1	−0.2 ± 0.9	2.7 ± 0.5	2.2 ± 0.2	−0.2 ± 0.2
**K**	−0.2 ± 0.3	0.3 ± 0.5	−0.9 ± 1.0	0.0 ± 0.5	0.8 ± 2.4	1.2 ± 0.5	3.0 ± 1.6	1.4 ± 1.0	1.0 ± 0.4	0.3 ± 0.3	−0.1 ± 0.7	2.8 ± 0.6	−0.2 ± 0.1	0.2 ± 0.7
**L**	−0.0 ± 0.0	0.6 ± 0.7	−0.7 ± 0.8	−0.3 ± 0.6	0.9 ± 2.2	0.4 ± 0.2	3.5 ± 2.5	1.4 ± 0.4	−0.7 ± 0.4	−0.6 ± 0.1	−0.1 ± 0.8	2.1 ± 0.5	−0.0 ± 0.1	0.1 ± 0.5
**M**	−0.2 ± 0.1	0.6 ± 0.9	−0.8 ± 0.8	−0.2 ± 0.6	0.2 ± 1.8	0.0 ± 0.3	1.6 ± 1.6	0.6 ± 1.0	−0.5 ± 0.4	−0.3 ± 0.1	−0.6 ± 0.6	1.9 ± 0.6	−0.1 ± 0.1	−0.2 ± 0.6
**N**	−0.2 ± 0.6	1.1 ± 0.7	−0.5 ± 0.8	0.4 ± 0.2	1.7 ± 1.5		4.4 ± 1.6	3.3 ± 0.7	1.5 ± 0.5			2.4 ± 0.5	−0.3 ± 0.1	1.1 ± 1.1
**P**	0.2 ± 0.2	1.9 ± 0.7	1.3 ± 2.4	−0.2 ± 0.9	2.1 ± 1.3	6.5 ± 2.5	1.0 ± 0.5	7.9 ± 0.9	2.6 ± 0.7	3.0 ± 0.9	5.2 ± 1.2	1.1 ± 0.5	3.3 ± 0.4	2.4 ± 0.0
**Q**	−0.1 ± 0.3	0.9 ± 0.7	−0.3 ± 0.9	−0.1 ± 0.3	1.3 ± 1.9	1.1 ± 0.4	3.5 ± 2.0	2.7 ± 0.7	1.2 ± 0.3	0.6 ± 0.3	0.6 ± 0.7	2.6 ± 0.6	−0.1 ± 0.1	0.7 ± 0.9
**R**	−0.4 ± 0.4		−0.8 ± 1.1	0.2 ± 0.5	1.1 ± 2.2	0.8 ± 0.1	3.7 ± 2.7		2.9 ± 0.8	0.8 ± 0.3	−0.2 ± 0.7	3.0 ± 0.8	−0.1 ± 0.1	0.2 ± 0.8
**S**	−0.2 ± 0.7	1.2 ± 0.7	0.1 ± 1.2	0.9 ± 0.3		1.9 ± 0.2	0.9 ± 0.3	2.9 ± 0.9	1.9 ± 0.3	0.3 ± 0.2	1.6 ± 0.8	2.2 ± 0.5	−0.2 ± 0.3	1.6 ± 1.0
**T**	−0.2 ± 0.6	1.3 ± 0.7	0.6 ± 0.4	1.1 ± 0.4	0.8 ± 0.9	1.2 ± 0.2	2.5 ± 1.4	3.2 ± 1.1	1.1 ± 0.0	0.3 ± 0.1	1.2 ± 1.0	2.8 ± 0.4	0.8 ± 0.3	1.3 ± 0.7
**V**	0.1 ± 0.1	1.2 ± 0.7	0.7 ± 0.5	0.8 ± 0.6	0.9 ± 1.3	1.6 ± 0.3	2.7 ± 1.1	2.5 ± 1.2		0.1 ± 0.1	0.1 ± 0.7	2.9 ± 0.4	1.7 ± 0.2	
**W**	−0.1 ± 0.4	1.0 ± 0.9	−0.2 ± 0.6	0.9 ± 0.5	6.0 ± 2.6	0.8 ± 1.3	16.5 ± 6.1	3.3 ± 2.0	16.2 ± 5.4	4.2 ± 1.5	1.1 ± 1.4	3.2 ± 0.8	0.1 ± 0.1	2.3 ± 2.6
**Y**	0.1 ± 0.4	0.7 ± 0.8	−0.4 ± 0.9	0.5 ± 0.2	4.3 ± 2.7	−0.1 ± 0.9	12.5 ± 5.0	3.8 ± 1.3	12.9 ± 4.6	1.2 ± 0.6	1.0 ± 1.5	2.6 ± 0.5	−0.0 ± 0.2	1.8 ± 2.4

[a] Standard deviation computed from three replicate simulations and mutational energies predicted from 100 frames extracted from the last 20 ns from each simulation.

Table S5: Mutational energies (kcal/mol) of gGID* at α4(+)β2(−) interface as predicted by FoldX 5.0.

AA	I1	R2	D3	E4	S7	N8	A10	R12	V13	N14	N15	O16	H17	V18
**A**	0.0 ± 0.1[a]	0.8 ± 0.1	−0.3 ± 0.1	−0.4 ± 0.1	−0.2 ± 0.1	2.3 ± 0.3		1.8 ± 0.2	1.3 ± 0.2	1.5 ± 0.2	1.2 ± 0.7	1.4 ± 0.5	0.4 ± 0.1	1.4 ± 0.4
**C**	−0.0 ± 0.1	0.8 ± 0.0	−0.2 ± 0.2	−0.5 ± 0.2	0.5 ± 0.2	2.2 ± 0.4	0.5 ± 0.2	2.0 ± 0.1	1.4 ± 0.2	1.3 ± 0.1	1.1 ± 0.4	1.4 ± 0.3	0.2 ± 0.2	1.1 ± 0.3
**D**	0.2 ± 0.1	1.4 ± 0.0		0.1 ± 0.1	5.3 ± 1.2	0.7 ± 0.5	2.8 ± 0.5	3.9 ± 0.3	2.6 ± 0.6	2.7 ± 0.2	3.2 ± 0.2	2.1 ± 0.8	0.4 ± 0.1	3.5 ± 0.6
**E**	0.1 ± 0.1	0.9 ± 0.1	−0.3 ± 0.3		7.5 ± 1.9	0.6 ± 0.6	1.0 ± 0.7	3.3 ± 0.3	2.3 ± 0.5	2.3 ± 0.1	2.5 ± 0.3	1.7 ± 0.6	0.1 ± 0.1	2.4 ± 0.8
**F**	0.1 ± 0.0	0.7 ± 0.1	−0.4 ± 0.2	−1.2 ± 0.1	13.6 ± 6.1	0.3 ± 0.5	6.9 ± 1.1	0.4 ± 0.3	2.4 ± 1.9	1.3 ± 0.2	0.9 ± 0.1	1.4 ± 1.1	−0.7 ± 0.1	3.6 ± 2.7
**G**	−0.1 ± 0.1	0.9 ± 0.1	0.2 ± 0.2	0.1 ± 0.4	0.6 ± 0.2	3.1 ± 0.4	1.4 ± 0.1	3.0 ± 0.2	2.2 ± 0.2	1.8 ± 0.1	2.8 ± 0.8	1.5 ± 0.6	0.6 ± 0.2	2.0 ± 0.5
**H**	−0.2 ± 0.0	0.6 ± 0.1	−0.3 ± 0.2	−0.7 ± 0.3	16.9 ± 6.8	1.7 ± 0.5	16.6 ± 6.2	2.1 ± 0.3	5.2 ± 2.8	2.4 ± 0.4	1.2 ± 0.3	2.1 ± 0.9		5.0 ± 3.7
**I**		1.0 ± 0.1	−0.1 ± 0.4	−0.5 ± 0.5	3.6 ± 1.4	3.4 ± 0.6	0.8 ± 0.7	2.0 ± 1.0	−0.3 ± 0.6	1.6 ± 0.1	1.0 ± 0.0	1.6 ± 0.3	−0.1 ± 0.1	0.4 ± 0.5
**K**	−0.4 ± 0.0	0.4 ± 0.1	−0.2 ± 0.1	−1.3 ± 0.2	5.6 ± 2.8	2.8 ± 0.5	0.8 ± 0.1	0.9 ± 0.2	0.8 ± 0.4	1.4 ± 0.2	0.0 ± 0.5	1.1 ± 0.7	−0.5 ± 0.1	0.9 ± 0.7
**L**	−0.1 ± 0.0	0.8 ± 0.1	−0.4 ± 0.1	−0.8 ± 0.2	4.8 ± 2.3	2.2 ± 0.6	1.1 ± 0.6	0.0 ± 0.1	−0.5 ± 0.4	0.5 ± 0.1	0.6 ± 0.2	1.1 ± 0.3	−0.3 ± 0.1	0.5 ± 0.5
**M**	−0.2 ± 0.0	0.8 ± 0.1	−0.6 ± 0.2	−1.1 ± 0.2	3.6 ± 1.9	0.8 ± 0.4	−0.7 ± 0.2	−0.0 ± 0.1	−0.9 ± 0.2	0.6 ± 0.0	0.2 ± 0.2	0.5 ± 0.2	−0.5 ± 0.1	0.5 ± 0.6
**N**	−0.1 ± 0.1	0.8 ± 0.0	−0.4 ± 0.3	−0.9 ± 0.3	3.5 ± 1.0		1.9 ± 0.5	2.4 ± 0.2	1.9 ± 0.2			1.3 ± 0.4	0.2 ± 0.1	2.0 ± 0.8
**P**	0.0 ± 0.0	1.7 ± 0.4	−0.7 ± 0.4	2.1 ± 1.6	1.1 ± 0.3	5.0 ± 0.7	0.1 ± 0.3	8.4 ± 2.2	2.5 ± 1.0	3.0 ± 0.7	5.2 ± 0.9	0.2 ± 0.1	1.5 ± 0.3	3.6 ± 1.5
**Q**	−0.2 ± 0.0	0.5 ± 0.1	−0.3 ± 0.1	−0.8 ± 0.2	5.5 ± 2.0	2.2 ± 0.6	0.9 ± 0.3	1.6 ± 0.3	1.5 ± 0.4	0.9 ± 0.1	1.1 ± 0.5	1.3 ± 0.6	−0.0 ± 0.1	1.6 ± 0.7
**R**	−0.8 ± 0.0		−0.4 ± 0.2	−1.4 ± 0.3	6.1 ± 3.4	2.5 ± 0.6	0.2 ± 0.1		2.3 ± 1.1	1.6 ± 0.2	−0.1 ± 0.4	0.8 ± 0.8	−0.5 ± 0.0	1.0 ± 0.7
**S**	−0.0 ± 0.1	0.8 ± 0.1	−0.3 ± 0.2	−0.3 ± 0.2		1.8 ± 0.3	0.7 ± 0.1	2.4 ± 0.1	2.1 ± 0.3	1.6 ± 0.2	1.8 ± 0.5	1.3 ± 0.5	0.4 ± 0.2	2.1 ± 0.7
**T**	0.0 ± 0.0	0.8 ± 0.1	0.0 ± 0.3	−0.2 ± 0.2	1.0 ± 0.5	1.5 ± 0.5	0.8 ± 0.4	2.7 ± 0.2	1.5 ± 0.1	1.8 ± 0.4	2.1 ± 0.5	1.9 ± 0.4	0.6 ± 0.2	1.7 ± 0.5
**V**	0.1 ± 0.0	1.0 ± 0.1	−0.1 ± 0.2	−0.3 ± 0.5	1.8 ± 1.0	2.1 ± 0.3	0.5 ± 0.4	2.2 ± 0.7		1.8 ± 0.2	1.3 ± 0.2	1.9 ± 0.4	0.3 ± 0.1	
**W**	−0.2 ± 0.1	0.9 ± 0.1	0.2 ± 0.1	−0.8 ± 0.6	22.3 ± 10.0	2.8 ± 0.8	7.7 ± 0.8	1.5 ± 1.2	6.7 ± 1.3	2.7 ± 0.7	1.8 ± 0.3	1.5 ± 1.0	−0.5 ± 0.1	4.6 ± 3.3
**Y**	0.1 ± 0.1	0.7 ± 0.1	−0.3 ± 0.1	−1.2 ± 0.2	16.1 ± 7.6	0.9 ± 0.5	7.6 ± 1.3	0.9 ± 0.3	4.2 ± 2.7	1.9 ± 0.6	1.2 ± 0.1	1.7 ± 0.8	−0.9 ± 0.1	4.2 ± 3.4

[a] Standard deviation computed from three replicate simulations and mutational energies predicted from 100 frames extracted from the last 20 ns from each simulation.

Table S6: Mutational energies (kcal/mol) of gGID* at $\alpha7(+)\alpha7(-)$ interface as predicted by FoldX 5.0.

AA	I1	R2	D3	E4	S7	N8	A10	R12	V13	N14	N15	O16	H17	V18
**A**	0.3 ± 0.4[a]	1.1 ± 0.7	0.5 ± 0.6	0.1 ± 0.5	0.2 ± 0.2	1.8 ± 0.1		1.5 ± 1.3	1.6 ± 0.1	1.2 ± 0.5	1.0 ± 0.5	2.2 ± 0.4	0.1 ± 0.1	1.2 ± 0.8
**C**	0.2 ± 0.3	1.2 ± 0.8	0.6 ± 0.4	0.1 ± 0.5	0.3 ± 0.2	1.2 ± 0.3	1.8 ± 0.5	1.9 ± 1.4	1.5 ± 0.1	1.1 ± 0.2	1.1 ± 0.6	2.4 ± 0.6	0.0 ± 0.1	0.8 ± 0.6
**D**	0.2 ± 0.2	1.7 ± 0.8		0.1 ± 0.1	2.1 ± 1.0	−0.4 ± 0.8	4.7 ± 0.5	4.0 ± 1.8	2.6 ± 0.7	2.6 ± 0.2	2.5 ± 0.9	2.7 ± 0.3	0.0 ± 0.1	1.5 ± 0.8
**E**	0.2 ± 0.5	1.1 ± 0.6	0.3 ± 0.6		1.8 ± 1.3	0.1 ± 0.3	3.5 ± 1.0	3.5 ± 1.3	2.4 ± 0.6	1.7 ± 0.5	2.4 ± 1.1	2.3 ± 0.2	−0.3 ± 0.1	1.0 ± 0.6
**F**	0.1 ± 0.2	0.9 ± 0.6	0.9 ± 1.3	−0.3 ± 0.9	0.5 ± 1.1	−0.4 ± 0.1	10.8 ± 0.7	1.4 ± 1.6	2.7 ± 1.4	1.6 ± 2.6	4.3 ± 7.3	2.2 ± 1.0	−0.8 ± 0.1	0.3 ± 0.5
**G**	0.2 ± 0.4	1.3 ± 0.9	0.8 ± 0.6	0.3 ± 0.5	0.4 ± 0.2	2.5 ± 0.2	1.3 ± 0.3	2.8 ± 1.4	2.3 ± 0.1	1.9 ± 0.2	2.1 ± 0.6	2.4 ± 0.7	0.4 ± 0.1	1.6 ± 1.2
**H**	0.2 ± 0.7	1.1 ± 0.8	2.3 ± 2.5	0.2 ± 0.7	1.2 ± 1.1	0.8 ± 0.1	39.9 ± 6.6	2.6 ± 0.4	4.6 ± 2.0	4.4 ± 4.7	7.3 ± 11.3	4.1 ± 1.3		1.3 ± 1.2
**I**		1.1 ± 0.4	0.6 ± 0.5	0.0 ± 0.5	1.0 ± 0.2	1.0 ± 1.1	4.3 ± 1.5	2.4 ± 2.0	−0.3 ± 0.4	2.0 ± 1.4	1.6 ± 1.2	2.5 ± 0.5	−0.4 ± 0.2	−0.1 ± 0.1
**K**	−0.2 ± 0.4	0.5 ± 0.2	0.3 ± 0.8	−0.5 ± 0.7	−0.1 ± 0.4	0.6 ± 0.2	2.2 ± 0.6	1.0 ± 0.9	1.0 ± 1.4	0.7 ± 0.4	1.2 ± 2.1	2.8 ± 1.0	−0.6 ± 0.1	0.1 ± 0.6
**L**	−0.1 ± 0.0	0.9 ± 0.5	0.2 ± 0.6	−0.3 ± 0.5	0.2 ± 0.3	−0.0 ± 0.1	3.4 ± 0.4	0.6 ± 1.1	−0.5 ± 0.1	0.1 ± 0.3	0.7 ± 1.0	2.0 ± 0.8	−0.6 ± 0.1	−0.1 ± 0.1
**M**	−0.3 ± 0.1	0.8 ± 0.5	0.1 ± 0.4	−0.4 ± 0.5	−0.1 ± 0.2	−0.1 ± 0.1	0.6 ± 0.5	0.2 ± 1.0	−0.3 ± 0.5	0.1 ± 0.1	0.6 ± 1.1	1.6 ± 0.8	−0.8 ± 0.0	−0.1 ± 0.3
**N**	0.1 ± 0.4	1.2 ± 0.7	0.7 ± 0.6	−0.1 ± 0.6	0.7 ± 0.2	0	4.1 ± 0.4	2.5 ± 1.2	1.8 ± 0.1			2.6 ± 0.8	0.1 ± 0.1	1.1 ± 0.8
**P**	0.5 ± 0.7	1.4 ± 0.5	0.0 ± 0.8	0.5 ± 0.7	0.8 ± 0.4	4.4 ± 0.8	1.5 ± 0.5	7.7 ± 2.0	3.2 ± 0.3	5.0 ± 1.7	5.7 ± 0.6	0.6 ± 0.1	1.1 ± 0.4	2.6 ± 0.5
**Q**	0.1 ± 0.5	0.9 ± 0.4	0.5 ± 0.7	−0.3 ± 0.5	0.5 ± 0.5	1.0 ± 0.1	2.8 ± 1.0	2.0 ± 0.9	1.5 ± 0.8	0.8 ± 0.2	1.7 ± 1.6	2.6 ± 0.7	−0.3 ± 0.1	0.6 ± 0.8
**R**	−0.3 ± 0.6		0.4 ± 0.9	−0.5 ± 0.8	−0.2 ± 0.4	0.5 ± 0.3	2.6 ± 1.9		2.4 ± 2.0	0.7 ± 0.3	1.1 ± 2.2	3.3 ± 1.2	−0.5 ± 0.1	0.2 ± 0.7
**S**	0.2 ± 0.5	1.2 ± 0.8	0.7 ± 0.6	0.0 ± 0.5		1.9 ± 0.2	1.0 ± 0.5	2.1 ± 1.3	2.2 ± 0.3	1.4 ± 0.4	1.9 ± 0.8	2.6 ± 0.6	0.2 ± 0.0	1.3 ± 1.1
**T**	0.1 ± 0.2	1.2 ± 0.6	0.8 ± 0.5	0.2 ± 0.5	0.6 ± 0.3	1.4 ± 0.5	2.9 ± 1.3	2.8 ± 1.5	1.4 ± 0.4	1.9 ± 0.3	2.3 ± 0.8	3.3 ± 0.6	0.4 ± 0.1	1.0 ± 0.7
**V**	0.2 ± 0.2	1.3 ± 0.5	0.8 ± 0.5	0.3 ± 0.5	1.0 ± 0.2	1.2 ± 0.6	2.9 ± 1.1	2.4 ± 1.9		2.1 ± 0.7	1.6 ± 1.0	2.8 ± 0.4	0.0 ± 0.1	
**W**	0.0 ± 0.4	1.3 ± 0.8	1.5 ± 1.6	0.0 ± 0.8	1.2 ± 0.9	0.6 ± 0.4	15.4 ± 2.6	2.9 ± 1.6	5.8 ± 3.3	3.1 ± 3.5	4.2 ± 5.8	3.1 ± 1.2	−0.5 ± 0.1	0.7 ± 0.6
**Y**	0.3 ± 0.3	1.0 ± 0.7	1.1 ± 1.4	−0.2 ± 0.9	0.5 ± 0.8	−0.1 ± 0.1	12.4 ± 1.2	2.2 ± 1.7	5.1 ± 1.9	2.4 ± 4.0	5.8 ± 9.0	2.8 ± 1.2	−0.6 ± 0.3	0.5 ± 0.6

[a] Standard deviation computed from three replicate simulations and mutational energies predicted from 100 frames extracted from the last 20 ns from each simulation.

Table S7: Mutational energies (kcal/mol) of rGID* at $\alpha3(+)\alpha3(-)$ interface as predicted by FoldX 5.0.

AA	I1	R2	D3	E4	S7	N8	A10	R12	V13	N14	N15	O16	H17	V18
**A**	0.7 ± 0.5[a]	1.5 ± 0.6	0.0 ± 0.1	0.2 ± 0.1	0.1 ± 0.2	1.6 ± 0.1		0.8 ± 0.5	1.7 ± 0.1	1.0 ± 0.6	0.6 ± 0.2	1.7 ± 0.1	−0.1 ± 0.3	0.1 ± 0.4
**C**	0.5 ± 0.3	1.8 ± 0.5	0.2 ± 0.1	0.2 ± 0.1	0.1 ± 0.3	1.6 ± 0.1	1.2 ± 0.2	1.3 ± 0.6	1.7 ± 0.1	1.1 ± 0.7	0.1 ± 0.2	2.0 ± 0.0	0.1 ± 0.2	0.2 ± 0.3
**D**	0.7 ± 0.3	2.2 ± 0.6		0.1 ± 0.1	0.8 ± 0.2	−0.6 ± 0.0	4.2 ± 0.2	4.5 ± 1.7	3.4 ± 0.2	1.7 ± 0.5	2.4 ± 0.1	1.1 ± 0.2	0.3 ± 0.3	0.3 ± 0.5
**E**	0.7 ± 0.4	1.6 ± 0.5	−0.2 ± 0.1		0.5 ± 0.1	0.3 ± 0.1	3.3 ± 0.3	3.2 ± 1.0	2.0 ± 0.2	1.9 ± 0.5	1.5 ± 0.3	1.1 ± 0.1	−0.4 ± 0.4	−0.2 ± 0.3
**F**	0.3 ± 0.3	1.5 ± 0.5	0.0 ± 0.2	−0.2 ± 0.1	−0.3 ± 0.1	0.0 ± 0.2	8.4 ± 1.5	7.3 ± 4.1	12.2 ± 1.2	0.5 ± 0.5	1.4 ± 1.2	1.6 ± 0.1	−1.1 ± 0.3	−0.4 ± 0.2
**G**	0.8 ± 0.7	1.7 ± 0.6	0.2 ± 0.0	0.3 ± 0.0	0.4 ± 0.2	2.3 ± 0.1	1.4 ± 0.0	1.8 ± 0.7	2.5 ± 0.1	1.5 ± 0.6	1.6 ± 0.1	1.6 ± 0.6	0.7 ± 0.3	0.5 ± 0.6
**H**	0.8 ± 0.3	2.1 ± 0.6	0.3 ± 0.1	0.2 ± 0.0	1.2 ± 0.4	1.3 ± 0.3	28.1 ± 2.0	13.2 ± 8.2	15.2 ± 1.1	2.3 ± 0.7	5.1 ± 0.4	2.1 ± 0.1		0.4 ± 0.4
**I**		1.8 ± 0.3	0.2 ± 0.1	−0.0 ± 0.3	−0.4 ± 0.6	0.8 ± 0.1	3.5 ± 0.6	1.6 ± 0.7	−0.8 ± 0.0	1.1 ± 0.6	0.9 ± 0.5	2.0 ± 0.1	−1.0 ± 0.1	−0.5 ± 0.1
**K**	0.0 ± 0.3	1.1 ± 0.4	−0.1 ± 0.2	−0.4 ± 0.1	−0.0 ± 0.1	0.9 ± 0.1	1.9 ± 0.5	0.7 ± 0.4	0.4 ± 0.2	0.9 ± 0.7	−0.3 ± 0.4	1.2 ± 0.1	−0.9 ± 0.5	−0.7 ± 0.2
**L**	−0.0 ± 0.1	1.2 ± 0.3	−0.1 ± 0.1	−0.3 ± 0.1	−0.6 ± 0.3	−0.1 ± 0.2	2.7 ± 0.5	0.7 ± 1.0	−0.9 ± 0.2	−0.2 ± 0.7	−0.6 ± 0.5	1.4 ± 0.1	−1.5 ± 0.1	−0.6 ± 0.1
**M**	−0.5 ± 0.3	1.2 ± 0.4	−0.0 ± 0.2	−0.2 ± 0.1	−0.5 ± 0.4	−0.3 ± 0.2	0.4 ± 0.4	−0.5 ± 0.5	−1.1 ± 0.1	−0.2 ± 0.7	−1.5 ± 0.3	1.1 ± 0.1	−1.8 ± 0.3	−0.9 ± 0.2
**N**	0.8 ± 0.5	1.7 ± 0.7	0.2 ± 0.1	0.2 ± 0.1	0.3 ± 0.3		3.4 ± 0.4	2.0 ± 0.8	2.2 ± 0.1			1.5 ± 0.4	0.2 ± 0.2	0.2 ± 0.4
**P**	1.0 ± 0.6	1.0 ± 0.8	−0.3 ± 0.1	1.6 ± 0.3	−0.4 ± 0.3	0.6 ± 0.3	0.4 ± 0.5	6.6 ± 0.3	4.4 ± 0.3	2.7 ± 0.2	6.0 ± 0.7	0.0 ± 0.0	−1.6 ± 0.2	0.3 ± 0.3
**Q**	0.5 ± 0.3	1.4 ± 0.5	−0.0 ± 0.1	−0.0 ± 0.1	0.1 ± 0.1	1.1 ± 0.2	2.6 ± 0.3	1.4 ± 0.5	1.1 ± 0.1	0.9 ± 0.7	0.2 ± 0.2	1.5 ± 0.0	−0.4 ± 0.4	−0.2 ± 0.2
**R**	0.2 ± 0.3		−0.0 ± 0.2	−0.3 ± 0.1	0.4 ± 0.1	0.3 ± 0.3	2.1 ± 0.5		4.0 ± 0.6	1.3 ± 0.7	−0.4 ± 0.3	1.2 ± 0.2	−0.5 ± 0.5	−0.7 ± 0.2
**S**	0.8 ± 0.5	1.5 ± 0.8	0.2 ± 0.0	0.1 ± 0.1		1.8 ± 0.1	1.2 ± 0.1	1.6 ± 0.4	2.4 ± 0.1	1.5 ± 0.7	0.8 ± 0.1	1.6 ± 0.3	0.6 ± 0.3	0.4 ± 0.5
**T**	0.7 ± 0.3	1.9 ± 0.6	0.3 ± 0.1	0.4 ± 0.1	0.2 ± 0.3	1.2 ± 0.2	2.3 ± 0.4	2.4 ± 0.6	1.4 ± 0.1	1.8 ± 0.9	0.7 ± 0.5	2.4 ± 0.0	0.6 ± 0.6	0.9 ± 0.3
**V**	0.5 ± 0.1	2.1 ± 0.3	0.3 ± 0.0	0.3 ± 0.3	−0.1 ± 0.6	1.3 ± 0.1	1.8 ± 0.5	1.5 ± 0.6		1.3 ± 0.8	0.8 ± 0.6	2.2 ± 0.1	−0.2 ± 0.3	
**W**	0.1 ± 0.2	2.0 ± 0.9	0.1 ± 0.1	0.2 ± 0.2	0.4 ± 0.2	0.1 ± 0.4	10.1 ± 1.0	6.2 ± 4.2	21.3 ± 1.9	2.4 ± 0.7	2.9 ± 1.1	2.0 ± 0.2	−0.5 ± 0.6	−0.1 ± 0.3
**Y**	0.3 ± 0.3	1.6 ± 0.5	0.1 ± 0.2	−0.1 ± 0.1	−0.0 ± 0.2	0.4 ± 0.2	9.4 ± 1.4	9.1 ± 4.9	18.0 ± 2.1	0.8 ± 0.6	2.7 ± 1.7	1.7 ± 0.1	−0.7 ± 0.3	−0.3 ± 0.2

[a] Standard deviation computed from three replicate simulations and mutational energies predicted from 100 frames extracted from the last 20 ns from each simulation.

Table S8: Mutational energies (kcal/mol) of rGID* at $\alpha 4(+)\alpha 4(-)$ interface as predicted by FoldX 5.0.

AA	I1	R2	D3	E4	S7	N8	A10	R12	V13	N14	N15	O16	H17	V18
**A**	−0.1 ± 0.0[a]	0.7 ± 0.2	−0.2 ± 0.1	−0.2 ± 0.1	0.2 ± 0.8	0.9 ± 0.1		1.1 ± 0.7	1.0 ± 0.3	0.5 ± 1.4	0.5 ± 0.2	2.4 ± 0.5	0.3 ± 0.2	0.8 ± 0.7
**C**	0.1 ± 0.2	0.8 ± 0.3	0.0 ± 0.1	0.0 ± 0.0	1.0 ± 1.7	1.0 ± 0.1	1.0 ± 0.5	1.4 ± 1.0	0.9 ± 0.2	0.7 ± 1.3	0.4 ± 0.3	2.5 ± 0.4	0.3 ± 0.1	0.6 ± 0.5
**D**	0.3 ± 0.5	1.3 ± 0.2		0.7 ± 0.2	6.0 ± 5.0	0.1 ± 0.2	3.6 ± 0.7	2.7 ± 0.9	1.6 ± 0.3	1.3 ± 0.6	0.8 ± 0.6	2.3 ± 0.6	−0.1 ± 0.1	1.5 ± 1.3
**E**	0.2 ± 0.5	0.8 ± 0.5	−0.2 ± 0.2		7.1 ± 6.0	0.1 ± 0.3	3.0 ± 0.9	2.4 ± 0.9	1.8 ± 0.5	1.5 ± 0.7	1.0 ± 1.3	2.2 ± 0.3	−0.1 ± 0.2	1.1 ± 1.1
**F**	0.1 ± 0.1	0.5 ± 0.1	−0.5 ± 0.2	−0.6 ± 0.2	15.2 ± 13.5	−0.5 ± 0.3	6.8 ± 0.7	0.6 ± 0.6	3.5 ± 5.0	1.2 ± 0.4	1.8 ± 4.5	2.1 ± 0.7	−0.6 ± 0.5	0.7 ± 1.5
**G**	−0.3 ± 0.2	1.0 ± 0.1	−0.1 ± 0.2	0.2 ± 0.2	1.1 ± 0.9	2.2 ± 0.2	1.3 ± 0.0	1.9 ± 0.7	1.6 ± 0.3	0.9 ± 1.1	1.5 ± 0.6	2.6 ± 0.7	−0.7 ± 0.6	1.2 ± 0.8
**H**	0.2 ± 0.3	0.9 ± 0.3	−0.1 ± 0.1	0.3 ± 0.1	20.0 ± 12.2	1.1 ± 0.6	28.5 ± 2.8	1.9 ± 0.5	6.5 ± 7.6	2.3 ± 1.0	3.9 ± 6.9	2.8 ± 0.5		2.3 ± 2.6
**I**		0.9 ± 0.1	0.1 ± 0.2	−0.5 ± 0.3	3.7 ± 4.4	0.3 ± 0.4	2.9 ± 2.0	1.5 ± 1.8	−0.0 ± 0.2	0.7 ± 0.8	0.8 ± 1.1	2.4 ± 0.3	0.7 ± 0.1	−0.1 ± 0.3
**K**	−0.2 ± 0.2	0.3 ± 0.3	−0.6 ± 0.1	−0.8 ± 0.1	6.1 ± 6.4	0.3 ± 0.1	1.7 ± 1.0	0.2 ± 0.3	0.9 ± 0.4	0.5 ± 0.9	0.2 ± 1.2	2.4 ± 0.8	−0.1 ± 0.3	0.3 ± 0.8
**L**	−0.0 ± 0.1	0.4 ± 0.1	−0.5 ± 0.2	−0.8 ± 0.1	3.6 ± 5.4	−0.8 ± 0.2	1.9 ± 1.2	0.3 ± 0.9	−0.4 ± 0.3	0.1 ± 1.1	−0.2 ± 0.5	1.9 ± 0.2	−0.3 ± 0.3	−0.3 ± 0.3
**M**	−0.2 ± 0.1	0.4 ± 0.2	−0.5 ± 0.2	−0.8 ± 0.1	3.1 ± 4.9	−0.9 ± 0.2	0.0 ± 0.6	−0.1 ± 1.1	−0.6 ± 0.3	0.1 ± 1.1	−0.7 ± 0.1	1.6 ± 0.2	−0.6 ± 0.4	−0.4 ± 0.5
**N**	0.0 ± 0.3	0.7 ± 0.3	−0.2 ± 0.2	0.2 ± 0.2	4.1 ± 4.5		2.6 ± 0.6	1.5 ± 0.8	1.4 ± 0.1			2.2 ± 0.0	−0.2 ± 0.3	0.9 ± 0.8
**P**	0.2 ± 0.2	2.3 ± 2.7	−0.7 ± 0.8	0.2 ± 0.6	1.0 ± 3.4	0.6 ± 1.9	1.3 ± 0.8	6.8 ± 0.3	1.5 ± 1.0	2.7 ± 2.4	4.3 ± 2.6	0.8 ± 0.7	1.9 ± 2.7	0.7 ± 1.6
**Q**	−0.1 ± 0.3	0.5 ± 0.1	−0.3 ± 0.1	−0.3 ± 0.0	5.2 ± 5.6	0.3 ± 0.2	2.2 ± 0.7	1.4 ± 0.7	1.1 ± 0.1	0.7 ± 0.8	0.3 ± 0.9	2.5 ± 0.4	0.0 ± 0.1	0.7 ± 0.8
**R**	−0.4 ± 0.2		−0.7 ± 0.1	−0.7 ± 0.1	8.7 ± 8.7	−0.1 ± 0.3	1.0 ± 0.5		2.0 ± 0.6	0.8 ± 0.8	0.4 ± 1.9	2.5 ± 0.8	−0.1 ± 0.3	0.5 ± 0.9
**S**	0.1 ± 0.3	0.8 ± 0.3	−0.1 ± 0.1	0.2 ± 0.2		1.7 ± 0.4	1.0 ± 0.3	1.4 ± 0.7	1.4 ± 0.5	0.6 ± 1.1	0.8 ± 0.6	2.4 ± 0.6	−0.0 ± 0.2	1.0 ± 0.7
**T**	0.2 ± 0.3	1.0 ± 0.2	0.3 ± 0.3	0.5 ± 0.3	1.5 ± 1.2	1.2 ± 0.5	2.2 ± 1.5	2.2 ± 0.9	1.0 ± 0.4	0.7 ± 0.8	0.9 ± 0.8	3.0 ± 0.5	0.7 ± 0.2	0.8 ± 0.5
**V**	0.2 ± 0.2	1.1 ± 0.1	0.3 ± 0.2	0.0 ± 0.2	1.8 ± 2.9	0.5 ± 0.3	1.7 ± 1.6	1.6 ± 1.3		0.8 ± 1.0	0.6 ± 0.5	2.9 ± 0.5	1.1 ± 0.3	
**W**	0.1 ± 0.2	0.9 ± 0.3	−0.4 ± 0.3	−0.1 ± 0.2	23.2 ± 20.5	0.2 ± 0.9	7.8 ± 1.1	0.6 ± 0.9	6.5 ± 7.4	2.5 ± 0.8	3.2 ± 6.0	2.8 ± 0.5	−0.1 ± 0.3	2.5 ± 2.7
**Y**	0.2 ± 0.1	0.6 ± 0.2	−0.4 ± 0.2	−0.4 ± 0.2	17.1 ± 14.7	−0.1 ± 0.3	8.5 ± 1.0	1.4 ± 0.4	6.5 ± 8.2	1.7 ± 0.6	2.2 ± 5.0	2.4 ± 0.6	−0.3 ± 0.4	1.4 ± 1.9

[a] Standard deviation computed from three replicate simulations and mutational energies predicted from 100 frames extracted from the last 20 ns from each simulation.

Table S9: Mutational energies (kcal/mol) of rGID* at $\alpha7(+)\alpha7(-)$ interface as predicted by FoldX 5.0.

AA	I1	R2	D3	E4	S7	N8	A10	R12	V13	N14	N15	O16	H17	V18
**A**	−0.3 ± 0.6[a]	0.9 ± 0.4	−0.2 ± 0.1	0.6 ± 0.0	0.5 ± 0.4	1.1 ± 0.1		0.6 ± 0.3	0.7 ± 0.4	0.9 ± 0.7	0.3 ± 0.8	1.6 ± 0.3	0.2 ± 0.5	0.6 ± 0.3
**C**	0.1 ± 0.4	1.2 ± 0.4	0.1 ± 0.1	0.7 ± 0.1	0.5 ± 0.2	1.0 ± 0.2	1.4 ± 0.1	1.1 ± 0.3	0.6 ± 0.3	0.8 ± 0.6	0.2 ± 0.6	2.0 ± 0.5	0.3 ± 0.5	0.2 ± 0.1
**D**	0.9 ± 1.4	1.9 ± 0.3		0.6 ± 0.2	2.3 ± 0.5	−0.5 ± 0.4	4.5 ± 0.5	2.9 ± 0.5	1.8 ± 0.7	1.2 ± 0.9	1.4 ± 1.5	1.4 ± 0.5	0.4 ± 0.4	0.4 ± 0.1
**E**	0.9 ± 1.4	1.7 ± 0.5	−0.4 ± 0.1		1.9 ± 0.6	−0.2 ± 0.5	2.9 ± 0.4	2.7 ± 0.6	1.4 ± 0.7	0.9 ± 0.7	1.3 ± 0.5	1.5 ± 0.7	−0.2 ± 0.5	−0.2 ± 0.2
**F**	0.4 ± 0.9	0.8 ± 0.5	−0.3 ± 0.1	0.5 ± 0.3	0.3 ± 0.7	−1.0 ± 0.3	9.1 ± 1.0	0.3 ± 1.4	0.7 ± 1.8	−0.1 ± 0.7	4.0 ± 3.4	1.6 ± 0.6	−0.9 ± 0.3	−0.0 ± 0.1
**G**	−0.4 ± 0.7	1.3 ± 0.5	−0.0 ± 0.1	0.9 ± 0.0	0.7 ± 0.1	2.2 ± 0.1	1.5 ± 0.1	1.9 ± 0.2	1.3 ± 0.5	1.0 ± 0.7	1.2 ± 0.5	2.0 ± 0.5	0.6 ± 0.1	0.9 ± 0.3
**H**	0.2 ± 0.6	1.3 ± 0.3	0.1 ± 0.1	0.7 ± 0.2	2.6 ± 3.0	0.8 ± 0.2	38.9 ± 2.5	2.2 ± 0.9	1.9 ± 1.6	0.7 ± 0.8	7.3 ± 7.0	2.2 ± 0.6		0.7 ± 0.1
**I**		1.4 ± 0.5	0.0 ± 0.1	0.7 ± 0.6	0.6 ± 0.2	0.9 ± 0.3	4.0 ± 0.3	1.2 ± 0.4	−0.1 ± 0.3	0.7 ± 0.6	0.4 ± 1.2	2.1 ± 0.7	−0.1 ± 0.6	−0.4 ± 0.2
**K**	−0.2 ± 0.3	0.6 ± 0.4	−0.4 ± 0.2	0.0 ± 0.1	0.5 ± 0.2	0.9 ± 0.1	2.3 ± 0.4	0.1 ± 0.4	−0.0 ± 0.2	0.1 ± 0.8	0.7 ± 2.0	1.6 ± 0.7	−0.3 ± 0.7	−0.4 ± 0.1
**L**	−0.1 ± 0.1	0.6 ± 0.5	−0.3 ± 0.2	−0.1 ± 0.2	0.3 ± 0.4	−0.2 ± 0.2	3.1 ± 0.6	0.1 ± 0.6	−0.7 ± 0.2	−0.1 ± 0.4	−0.3 ± 1.0	1.5 ± 0.5	−0.8 ± 0.8	−0.4 ± 0.1
**M**	−0.2 ± 0.1	0.5 ± 0.5	−0.2 ± 0.2	0.0 ± 0.4	0.0 ± 0.1	−0.6 ± 0.2	0.3 ± 0.2	−0.2 ± 0.5	−1.0 ± 0.1	−0.1 ± 0.4	−0.7 ± 0.9	1.4 ± 0.6	−1.1 ± 0.4	−0.5 ± 0.3
**N**	0.0 ± 0.7	1.1 ± 0.4	−0.0 ± 0.1	0.5 ± 0.2	0.8 ± 0.1		3.9 ± 0.4	1.4 ± 0.4	0.9 ± 0.5			1.9 ± 0.5	0.2 ± 0.0	0.2 ± 0.1
**P**	0.6 ± 1.0	2.1 ± 3.5	−1.1 ± 0.2	1.3 ± 1.1	−0.0 ± 0.7	−0.3 ± 0.3	1.4 ± 0.1	5.6 ± 1.0	0.8 ± 1.0	3.7 ± 1.2	4.9 ± 1.4	0.0 ± 0.2	−1.0 ± 0.5	1.3 ± 1.0
**Q**	0.3 ± 0.8	1.0 ± 0.5	−0.3 ± 0.1	−0.3 ± 0.3	0.8 ± 0.3	0.6 ± 0.1	2.3 ± 0.5	1.3 ± 0.6	0.5 ± 0.3	0.4 ± 0.6	1.0 ± 1.3	1.8 ± 0.7	−0.0 ± 0.5	−0.0 ± 0.1
**R**	−0.4 ± 0.3		−0.4 ± 0.1	0.2 ± 0.2	0.7 ± 0.7	0.6 ± 0.2	1.4 ± 0.8		1.0 ± 1.4	−0.0 ± 0.6	1.1 ± 2.8	1.9 ± 0.8	0.3 ± 0.7	−0.2 ± 0.1
**S**	0.1 ± 0.5	1.2 ± 0.4	−0.1 ± 0.1	0.8 ± 0.1		1.6 ± 0.1	1.1 ± 0.1	1.2 ± 0.3	1.1 ± 0.4	0.8 ± 0.8	0.9 ± 0.5	1.8 ± 0.4	0.6 ± 0.2	0.6 ± 0.3
**T**	0.2 ± 0.5	1.4 ± 0.5	0.2 ± 0.2	1.3 ± 0.3	0.4 ± 0.1	1.3 ± 0.2	3.0 ± 0.1	1.8 ± 0.2	0.9 ± 0.5	1.0 ± 1.0	0.7 ± 0.6	2.6 ± 0.6	0.8 ± 0.8	0.5 ± 0.4
**V**	0.2 ± 0.2	1.6 ± 0.4	0.2 ± 0.1	1.2 ± 0.5	0.7 ± 0.5	1.2 ± 0.2	2.7 ± 0.1	1.1 ± 0.3		1.1 ± 0.8	0.0 ± 0.7	2.1 ± 0.6	0.2 ± 0.9	
**W**	0.5 ± 1.0	1.2 ± 0.5	−0.1 ± 0.1	0.6 ± 0.4	1.9 ± 2.0	−0.1 ± 0.2	11.7 ± 1.8	2.5 ± 1.3	2.5 ± 2.2	0.4 ± 0.8	5.6 ± 3.9	2.1 ± 0.7	−0.2 ± 0.2	0.4 ± 0.2
**Y**	0.4 ± 0.9	0.9 ± 0.4	−0.2 ± 0.1	0.5 ± 0.3	1.0 ± 1.3	−0.7 ± 0.3	11.2 ± 1.0	0.8 ± 1.4	2.1 ± 3.0	0.1 ± 0.9	5.8 ± 3.3	1.8 ± 0.6	−0.2 ± 0.5	0.1 ± 0.2

[a] Standard deviation computed from three replicate simulations and mutational energies predicted from 100 frames extracted from the last 20 ns from each simulation.
